# A systematic review of the risk factors for clinical response to opioids for all-age patients with cancer-related pain and presentation of the paediatric STOP pain study

**DOI:** 10.1186/s12885-018-4478-3

**Published:** 2018-05-18

**Authors:** Ersilia Lucenteforte, Laura Vagnoli, Alessandra Pugi, Giada Crescioli, Niccolò Lombardi, Roberto Bonaiuti, Maurizio Aricò, Sabrina Giglio, Andrea Messeri, Alessandro Mugelli, Alfredo Vannacci, Valentina Maggini

**Affiliations:** 1 Department of Neuroscience, Psychology, Drug Research and Children’s Health, University of Florence, Florence, Italy; 2 Department of Clinical and Experimental Medicine, University of Pisa, Pisa, Italy; 3 Pain and Palliative Care Unit, Meyer children’s hospital, Florence, Italy; 4 Clinical Trial Office, Meyer Children’s Hospital, Florence, Italy; 5 Direzione Generale, Azienda Sanitaria Provinciale, Ragusa, Italy; 6 Medical Genetics Unit, Meyer Children’s University Hospital, Florence, Italy; 7 Medical Genetics Unit, Department of Clinical and Experimental Biomedical Sciences “Mario Serio”, University of Florence, Florence, Italy; 8 Center for Integrative Medicine, Careggi University Hospital, Department of Experimental and Clinical Medicine, University of Florence, Largo Brambilla, 3 - 50134 Florence, Italy

**Keywords:** Cancer pain, Children, Opioid efficacy, Opioid safety

## Abstract

**Background:**

Inter-patient variability in response to opioids is well known but a comprehensive definition of its pathophysiological mechanism is still lacking and, more importantly, no studies have focused on children. The STOP Pain project aimed to evaluate the risk factors that contribute to clinical response and adverse drug reactions to opioids by means of a systematic review and a clinical investigation on paediatric oncological patients.

**Methods:**

We conducted a systematic literature search in EMBASE and PubMed up to the 24th of November 2016 following Cochrane Handbook and PRISMA guidelines. Two independent reviewers screened titles and abstracts along with full-text papers; disagreements were resolved by discussion with two other independent reviewers. We used a data extraction form to provide details of the included studies, and conducted quality assessment using the Quality Assessment Tool for Observational Cohort and Cross-Sectional Studies.

**Results:**

Young age, lung or gastrointestinal cancer, neuropathic or breakthrough pain and anxiety or sleep disturbance were associated to a worse response to opioid analgesia. No clear association was identified in literature regarding gender, ethnicity, weight, presence of metastases, biochemical or hematological factors. Studies in children were lacking. Between June 2011 and April 2014, the Italian STOP Pain project enrolled 87 paediatric cancer patients under treatment with opioids (morphine, codeine, oxycodone, fentanyl and tramadol).

**Conclusions:**

Future studies on cancer pain should be designed with consideration for the highlighted factors to enhance our understanding of opioid non-response and safety. Studies in children are mandatory.

**Trial registration:**

CRD42017057740.

**Electronic supplementary material:**

The online version of this article (10.1186/s12885-018-4478-3) contains supplementary material, which is available to authorized users.

## Background

Worldwide incidence of childhood cancer is about 160,000 new cases/year with 90,000 deaths/year under 15 years of age [1]. Young people with cancer experience multiple symptoms which negatively affect their quality of life [2]. Children with cancer often report pain (up to 89% of patients in an advanced stage of the disease) and over 70% of them sometimes report severe pain [3, 4]. Even though pain relief is one of the main concerns of physicians [5] and the inter-patient variability in response to opioids is well known [6], pain relief is still often misdiagnosed or treated inappropriately. In adults, current evidence suggests that several factors may influence analgesic response during the course of the illness [7]. For example, it has been reported that men require more morphine in the postoperative period than women [8] and obesity may partly explain inter-individual variations in opioid efficacy and toxicity [9]. Moreover, it is fundamental to consider the influence of genetic factors regulating opioid pharmacokinetics (e.g. UDP-glucuronosyltransferase genes, *UGT*) [10] and pharmacodynamics (e.g. μ-opioid receptor gene, *OPRM1*) [11] on opioid response variability. In this frame, the region of Tuscany (Italy) developed a research program (“Pharmacogenetics in pain therapy”) in 2006 to evaluate the association between single nucleotide polymorphisms (SNPs) in metabolizing genes and the response to opioids in a general population. To the best of our knowledge, no similar epidemiological and genetic study has yet been conducted to address these important issues in paediatric populations even if physiological differences between adults and children are well known [12]. Furthermore, children experience illnesses and are subjected to medical care differently from adults and they depend on their parents to cope with stressful situations [13]. Therefore, familiar context may be an influencing factor on the perception of pain and the efficacy of pain therapy.

For these reasons, in 2010 we designed a longitudinal study focused only on paediatric cancer patients, called STOP Pain (Suitable Treatment for Oncologic Paediatric Pain) to continue recruitment for the regional study in an attempt to get as homogeneous a sample as possible, i.e. patients from the same population (with cancer pain) and treated in a homogeneous way. The main objectives of the project are to conduct a comprehensive literature review of the association between inter-individual opioid responsiveness, socio-demographic and medical factors and to evaluate the risk factors that contribute to response/non-response and adverse drug reactions to opioids in a sample of paediatric oncological patients.

## Methods

### Systematic review of current literature

This review was performed in accordance with the Cochrane Handbook and the Prisma Statement for Systematic Reviews [14] and it was registered in PROSPERO with the number CRD42017057740 [15].

A systematic PUBMED and EMBASE search for any study evaluating opioid non-response and safety among cancer patients was performed up to the 24th of November 2016. Four themes (drugs, cancer, randomized clinical trials, and observational studies) were combined by using the Boolean operator “and” (see full search strategy in Additional file 1). We took into consideration articles (excluding letters) published in English and Italian, and studies on humans using the corresponding filters. We also searched the papers among those quoted as references in the retrieved studies, as well as in a few previous reviews.

Two investigators (AP and GC) independently reviewed titles and abstracts, and selected articles. Any disagreements was resolved through discussion and consensus with two other independent reviewers (EL and VM).

In a second phase, we retrieved the full texts and selected the original articles based on the following criteria:

1. Patients included were cancer patients
2. Drugs involved were opioids
3. Outcomes evaluated were opioid non-response and safety (see Additional file 2)
4. One or more variables were studied as factors associated to therapy outcome

We decided not to consider putative genetic factors since a large body of evidence was already available. Moreover, we excluded pharmacokinetic studies as well as clinical trials and comparative studies evaluating different drugs, drug doses, formulations and administration routes.

For each retrieved study, we extracted the following data: location, year of publication, study type, size, mean age and gender of the sample, tumor characteristics, drugs used and main findings.

The quality of the included studies was assessed using the “Quality Assessment Tool for Observational Cohort and Cross-Sectional Studies” [16] following the criteria reported in Additional file 3.

### STOP pain project

The study enrolled paediatric patients receiving opioids (morphine, codeine, oxycodone, fentanyl and tramadol) for cancer-related pain relief between June 2011 and April 2014. The institutional review board of Meyer Children’s Hospital approved the study.

Two structured questionnaires were administered to the enrolled children or their parents after obtaining written informed consent. The first questionnaire included demographic information (e.g. age, gender, weight, height, and allergies), medical history, concomitant illnesses and lifestyle of the children. Data concerning cancer diagnosis and evolution of the disease were collected from medical records. Data on health conditions as well as other parameters potentially predictive of high or low treatment responsiveness were collected carefully with the aim of adjusting for any confounding variables and/or effect modifiers. The second questionnaire included demographic information on parents and family environment.

An Individual Case Report Form (CRF) recorded all data. Unique Patient Code anonymized the patient before the matching with genetic data. Peripheral blood or mouth swab (when possible) were collected after patient recruitment. DNA was isolated using EZ1 Extractor (Qiagen) and standard commercial kits. DNA concentration and purity was then measured with NanoDrop 2000 (Thermo Scientific) and stored at − 20 °C. Genotyping was carried out using the Taqman assay (ABI, Applied Biosystems, Foster City, CA). Taqman probes were designed and synthesized by Applied Biosystems, who also provided standard PCR profile and reaction conditions. PCR plates were read on a 7500 Fast Real Time PCR system (Applied Biosystems).

Opioid dosing was standardized through the conversion to intravenous (IV) morphine equivalents (ME) according to the following opioid equi-analgesic calculation [17]: IV ME = oral oxycodone*2/3 = IV tramadol*10 = oral tramadol*30 = oral codeine *30 = IV fentanyl/100. When the direct conversion factor to IV ME was not available, the dosage was first converted to oral morphine equivalents and then to IV ME (3:1). Data are presented as mean and standard deviation.

We considered the following two outcomes regarding dose: dose (mg/kg) of IV ME administered during the first 24 h of treatment (Dose_24h_) and total dose (mg/kg) of IV ME (Dose_tot_).

The Visual Analogue Scale (VAS) was compiled by children of older age (> 6 yrs). Wong & Baker FACES Pain Rating Scale was administered to children between 4 and 6 years of age as an alternative outcome measure. When self-reporting of pain was not possible, such as in children who had difficulty verbalizing the presence or intensity of pain, the FLACC scale was used. Nurses administered the scales for pain intensity to patients at the first examination, repeating the procedure eight-hourly for intra-individual pain intensity evaluation.

We considered the following three outcomes regarding pain intensity: pain intensity before treatment (PI_to_); difference between pain intensity after 24 h of treatment and PI_to_ (∆ _VAS_); time to reach the lowest possible pain intensity (Time_tot_).

Evaluated side effects were gastrointestinal effects (nausea/vomiting, diarrhea and constipation), central nervous system effects (agitation, drowsiness, headache and sedation), and all adverse effects (gastrointestinal and central nervous system effects, and itching).

We checked data for consistency and completeness by tabulating the variables of interest. We compared differences for mean values of continuous response variables by one-way ANOVA and differences for percentages of categorical variables by chi-square test.

## Results

### Systematic review

**Fig. 1:**
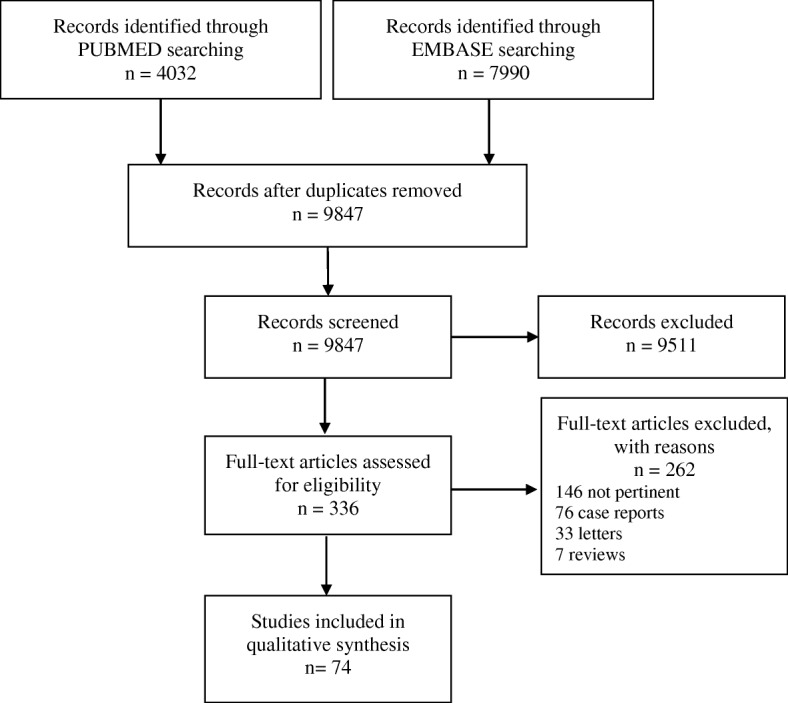
PRISMA Flow diagram

The PUMBED and EMBASE search produced 9847 records. A review of titles and abstracts resulted in the selection of 336 records of original studies, among which 72 met the inclusion criteria. Figure 1 reports the flowchart of study selection.

The characteristics of 74 studies included in this review [18–91] are reported in Additional file 2. Thirty-three studies were conducted in Europe, 16 in Asia, 22 in North America, one in South America (Brazil) and two in Turkey. Eighteen studies were published before 2000; 61 studies were conducted on more than 50 patients; two studies were conducted on female patients, two others mainly on males, and in the remaining studies the proportions of the two genders were similar. Four studies were conducted on children. The studies reported a broad variety of opioid non-response outcomes, and only a few outcomes were evaluated in more than two trials. In particular, non-response was defined as high dosage or pain intensity, low pain control or relief, switching, high Opioid Escalation Index (OEI) percentage, or worsening of pain. Patients used more than one opioid or non-specified opioids in 41 studies. Morphine was used alone in 19 studies, oxycodone alone in six, methadone alone in three and fentanyl alone in four studies. More than half of the included studies (41 out of 74) reported the opioid doses in milligrams of morphine equivalents.

For the included studies, the assessment of methodological quality was performed **(**Additional file 4**)** using the Quality Assessment Tool for Observational Cohort and Cross-Sectional Studies [16]. Items 1, 4, 6, 7, 9, 10, 11 and 13 have received affirmative responses from 100% of the studies included in the analysis. More than 50% of studies clearly defined the population analyzed (item 2) and performed an adjusted statistical analysis for confounding variables (item 14). Not reported (NR) was the answer to items 3 and 5 respectively in 58 and 99% of included studies, while not applicable (NA) was assigned for more than 50% of studies for items 8 and 12.

Given the high number of factors considered, we decided to report only the results on associations investigated in more than three studies and found in 39 studies including more than 50 subjects and published after 2000 (Table 1). With regard to socio-demographic factors, while no association was found with age in nine studies, nine other studies reported an inverse association (i.e. older patients had a better response compared to younger ones) while three found a direct one. Gender was not related to non-response in 15 studies, two studies reported that males had a worse response than females, while one study stated that they had a better one. Body mass index (BMI) was investigated in two studies with no notable relationship found, while a direct association between non-response and weight gain was reported in one study (i.e. patients with high weight had a worse response compared to low-weight patients). Finally, two studies reported no association with alcoholism and one a direct association with alcohol abuse (i.e. subjects who abused alcohol had a worse response compared to those with normal drinking habits).

**Table 1:** Association between investigated factors and opioid non-response reported in 39 studies included in the systematic review

Factors	Association [reference]		
	None	Direct	Inverse
Sociodemographic			
Age (advanced)	[22, 24, 25, 30, 36, 50, 51, 61, 77]	[53, 63, 83]	[38, 40, 57, 58, 65, 66, 78, 80, 81]
Gender (male)	[24, 25, 36, 38, 40, 47, 51, 57, 58, 61, 65, 66, 77, 78, 86]	[53, 83]	[22]
Weight/body mass index	Body mass index [51, 58]	Weight gain (higher or equal to 1 kg) [25]	
Alcohol	Alcoholism [28, 72]	Abuse [56]	
Clinical			
Cancer diagnosis	[23, 24, 36, 38, 51, 58, 62, 77, 83]	Gastrointestinal, lung and ovarian [78]; mesothelioma [41]; lower gastrointestinal tract [25]; breast [53]; head and neck and lung [50]; tongue [46]	Non sex-related cancers [57]
Biochemical parameters	[35]; Serum creatinine and total bilirubin [53]; sodium, calcium, albumin, creatinine [25]	Alanine aminotransferase [53]; white blood cells count [25]	Total proteins [53]; vitamin D levels [87]
Cognition (poor)	[51, 65, 66, 88]	[48, 83, 85]	[22]
Metastasis location	Liver [51]; skeletal [24]	Bone [77, 78]; brain [78]; spinal [81]	Bone [22]; lung [81]
Psychological distress	[29, 88]; Mood (local questionnaire sad/depressed) [77]; depression (HAD) [22]	EORTC functioning [65, 66]; emotional non-well-being (FACT-G) or anxiety (MHI) [56]; anxiety (preop STAI) or Depression levels (preop BDI) [30]	EORTC functioning [51]
Sleep disturbance	[77]	[50, 51]	[22]
Pain related			
Breakthrough cancer pain (presence vs absence)	[22]	[51, 59]	
Incidental (presence vs absence)	[50, 88]	[65, 66]	
Intensity	Self-reported δ VAS [58]	Baseline-intervals [50]; initial pain intensity [83]	Initial worst pain severity [56]
Pathophysiology (neuropathic pain)	[35, 38, 43, 51, 58, 78]	Presence vs absence [24, 88]; neuropathic versus nociceptive [65, 66]	Presence vs absence [22]
Drug use			
Analgesics	[72]; NSAIDs [50]; previous [24]	Ketorolac [62]; NSAIDs [78]	
Adjuvants	[35]; Anticonvulsants [22]; gabapentin [51]; antidepressants (vs no antidepressant) [78]	Steroids or non-opioids [51]; steroids [78]; traditional sedatives [81]	
Others	Oncologic treatments [24]; neuromuscular blockade [62]	Antiemetics and beta blockers [25]	PPIs and recent chemotherapy (14 days) [25]; Prophylactic laxative treatments [63]

*BDI* Beck Depression Inventory, *EORTC* European Organization for Research and Treatment of Cancer, *FACT-G* Functional Assessment of Cancer Therapy-General, *HAD* Hospital Anxiety and Depression, *MHI* Mental Health Inventory, *MMSE* Mini Mental State Examination, *NSAID* Non-Steroidal Anti-Inflammatory Drug, *PPI* Proton-pump Inhibitor, *STAI* State-Trait Anxiety Inventory, *VAS* Visual Analog Scale

Cancer diagnosis, biochemical parameters, cognition, metastases location, psychological distress, and sleep disturbances were the clinical factors studied, with high heterogeneity of specific measures investigated and no clear association with outcomes. However, patients with lung cancer or mesothelioma (three out of 16 studies) or gastrointestinal cancer (two studies) had a worse response (direct association), as did patients with anxiety (two studies), or sleep disturbance (two out of four studies).

Pain-related factors were investigated and two studies reported a direct association between breakthrough cancer pain and worse response (i.e. subjects with breakthrough pain had a worse response), while one study reported no association. Similarly, two studies reported a direct association with incidental pain while two others found no association.

High heterogeneity emerged for measures of pain intensity and with regard to pain pathophysiology (neuropathic, visceral, somatic, etc.) with no clear association, although four out of 11 studies reported a direct association between the presence of neuropathic pain and opioid non-response.

Finally, analgesic drugs, adjuvants and other drugs were often studied as possible factors associated with non-response but contrasting results were reported.

Results on predictive factors of opioid safety are shown in Table 2.

**Table 2:** Association between investigated factors and opioid side effects reported in 9 studies included in the systematic review

Factors	Association [reference]		
	None	Direct	Inverse
Sociodemographic			
Age (advanced)	Anorexia, itch, nausea [61]; constipation [40, 67]; confusion, drowsiness [40]	Constipation, dry mouth, hallucination [61]	Myoclonus and urinary hesitancy [61]; dry mouth, nausea [40]
Gender (female)	Constipation [67]; itch, myoclonus, nausea, urinary hesitancy [61]	Anorexia, dry mouth [61]	
Clinical			
Biochemical factors	Constipation [67]		
Glomerular Filtration Rate (GFR; low vs normal)	Fatigue, nausea and vomiting, pain, cognitive dysfunction [84]	Loss of appetite, constipation [84]	
Cancer diagnosis	Constipation [67]		
Comorbidity (Charlson score)		Constipation [74]	
Terminal stage (decreasing weeks before death)	Nausea, vomiting [40]		Confusion, dry mouth, drowsiness, constipation [40]
Pain related			
Neuropathic pain (vs absence)		Dry mouth, confusion [40]	
Visceral pain (vs somatic pain)		Nausea, vomiting, constipation, dry mouth [40]	Confusion [40]
Drug use			
Co-use of ≥2 opioids		Constipation [74]	
Dose (high)	ADR [38]; constipation [67, 77, 78]; nausea [61, 77, 78]; dizziness, myoclonus [61]; dry mouth, emesis [78]; anorexia, somnolence, vomiting [77]	Urinary hesitancy [61]	Dry mouth [77]
Length of therapy (days)	Constipation [67]	Constipation [74]	
Switching		Dysuria [37]; constipation [74]	

Compared to younger patients, older ones reported a lower rate of myoclonus, urinary hesitancy, dry mouth, and nausea. No notable relationship was found between gender and itch, myoclonus, nausea, urinary hesitancy and constipation nor between age and anorexia, itch, nausea, constipation, confusion, or drowsiness, whereas anorexia and dry mouth were more common in female patients and constipation, dry mouth, and hallucinations were more frequent in older patients in only one study. We found no relationship between most clinical factors (i.e. biochemical parameters, cancer diagnosis and terminal stage) and the occurrence of adverse events although constipation was related to low glomerular filtration rate (GFR), comorbidity and terminal stage, as well as to confusion and drowsiness. The presence of neuropathic or somatic pain was related to the onset of confusion and dry mouth; while visceral pain was associated with a high frequency of dry mouth and gastrointestinal symptoms.

No significant differences in side effects were observed regarding anorexia, somnolence, nausea, vomiting, constipation, dry mouth, and emesis despite high opioid doses. However, an inverse association was found between opioid dose and dry mouth, while one study reported a direct association with urinary hesitancy. One study found a direct association between the length of opioid therapy and concomitant use of more than two opioids and the onset of constipation; nevertheless, the association was denied in another study. A direct relationship was found between opioid switching, dysuria, and constipation.

Validation of the Prisma checklist for the systematic review is reported in Additional file 5.

### STOP pain project

One hundred twenty-nine (75 + 54) patients met the inclusion criteria and resulted eligible for the study. Informed consent was requested from parents of 54 children but they were not included in the study for the following reasons: refusals (*n* = 6), terminally ill patients (*n* = 9), early discharge (*n* = 7), hospitalization in sterile room (n = 7), non-Italian speaking parents (*n* = 25).

The data set consisted of the characteristics of 87 patients enrolled between June 2011 and April 2014. For seven patients the biological sample was not available. STAI test was completed by 40 parents (37 mothers).

Table 3 presents the distribution of selected characteristics among 87 cancer patients included in the STOP Pain Project. The majority of children were male (56.32%) with more than 3 years of age (36.78% between 3 and 12 years, 42.53% over 12), and a BMI of more than 15 kg/m^2^ (44.83% between 15 and 20 kg/m^2^, 25.29% more than 20). Cancer diagnoses were mainly leukemia and lymphoma (39.08), sarcoma (20.69%) or osteosarcoma (19.54%), in 26.44% of cases with metastases, with oral cavity (49.43%) or skeletal (16.09%) pain. Patients were treated with morphine (68.97%), tramadol (21.84%), oxycodone (2.30%), codeine (2.30%) and more than one opioid (4.60%) for the achievement of pain relief. Table 4 shows the selected outcomes to evaluate opioid responsiveness of the 87 patients in terms of opioid dosage requirements and pain intensity assessment.

**Table 3:** Demographic and clinical characteristics of 87 patients

	Number	Percent
Gender		
Male	49	56.32
Female	38	43.68
Age (months)		
0–36	18	20.69
> 36–144	32	36.78
> 144	37	42.53
BMI		
< 15	25	28.74
15- < 20	39	44.83
≥ 20	22	25.29
*missing*	1	1.15
Diagnosis		
Brain Tumor	6	6.90
Leukemia and Lymphoma	34	39.08
Neuroblastoma	6	6.90
Osteosarcoma	17	19.54
Sarcoma	18	20.69
Others	6	6.90
Metastasis		
No	64	73.56
Yes	23	26.44

**Table 4:** Pain intensity assessment and opioid dosage requirements of the 87 patients

	mean ± SD	
PI_t0_ (Pain Intensity at t_0_)	4.34 ± 2.17	
PI_24h_ (Pain intensity at t_24h_)	2.04 ± 2.56	
PI_end_ (Pain intensity at t_end_)	1.07 ± 2.19	
Time_tot_ (time to the minor PI)	140.43 ± 63.89	
	N	%
PI_t0_ (grouped)		
≤4	47	54.02
> 4	40	45.98
Δ_VAS_ (PI_t0_ - PI_t24h_; grouped)		
≤ 2	46	52.87
> 2	39	44.83
Responders (PI_end_ equal to 0)		
No	23	26.44
Yes	64	73.56
Pain location		
Abdominal	12	13.79
Oral cavity	43	49.43
Skeletal - Muscle	14	16.09
Other	18	20.69
Drug		
morphine	60	68.97
tramadol	19	21.84
oxycodone	2	2.30
codeine	2	2.30
more than one	4	4.60
Dose_24h_ (mg/kg)		
≤ 0.2	24	27.59
0.2- ≤ 0.42	20	22.99
0.42- ≤ 0.50	22	25.29
> 0.50	19	21.84
*Missing*	2	2.30
Dose_tot_ (mg/kg)		
≤ 1.2	21	24.14
1.2- ≤ 2.16	20	22.99
2.16- ≤ 3.42	24	27.59
> 3.42	22	25.29

*PI* Pain Intensity

The aim of the present study was to investigate patient’s genetic predisposing trait (single nucleotide polymorphisms of genes involved in opioid transport, target and metabolism) to opioid responsiveness and safety profile. The investigated genes were ***ABCB1*** (ATP binding cassette subfamily B member 1), ***COMT*** (catechol-O-methyltransferase), ***IL6*** and ***IL8*** (interleukin 6 and 8), ***KCNJ6*** (potassium inwardly-rectifying channel, subfamily J, member 6), ***NR1I2*** (nuclear receptor subfamily 1 group I member 2), ***OPRM1*** (opioid receptor, mu 1), ***TNF-α*** (tumor necrosis factor α) and ***UGT2B7*** (UDP glucuronosyltransferase 2 family, polypeptide B7). For SNP selection, high priority was given to those SNPs for which functional alteration data were available in the literature and the minor allele frequency is above 15% in the Caucasian population.

## Discussion

### Systematic review

The large inter-individual variability in response to opioid analgesia and high prevalence of adverse events associated with their use underline the clinical importance of being able to predict who will or will not respond to opioid treatment.

#### Patient characteristics

According to our review, opioid non-response is associated with age in that older patients had a better response (inverse association). This result can be found in the majority of studies that show that elderly patients present an increased sensitivity to opioids [40]. Indeed, as age increases, there is a decrease in the volume of distribution and clearance of morphine, as well as a decrease in plasma albumin--the latter resulting in a greater unbound fraction of drug. These pharmacokinetic factors will lead to higher plasma levels and a longer duration of morphine action in elderly compared to younger patients receiving the same dose of the drug. In contrast with the widely reported sex-related differences in opioid response [92], we found no influence of gender. In particular, according to literature, females are more sensitive to morphine than males [93] while pain perception is reported to increase with the lowering of estrogen levels (such as in menopause) [94], suggesting that aging might contribute to level the gender differences in opioid response. This could explain the lack of association between gender and non-response found in our review since all included studies enrolled patients over the age of 60 and did not take into account menopausal status.

#### Type of cancer

We also found that patients with lung or gastrointestinal cancer had a worse response to opioid analgesia (direct association). Most surveys on cancer pain have not assessed the effect of primary diagnosis on the incidence, intensity, and treatment of cancer pain since the assessment of these effects is often complicated by the existence of multiple medical problems. The current evidence emerging mainly from studies conducted in palliative care units suggests that somatic pain is associated with lung, head and neck, breast, and prostate cancer, while visceral pain is associated with colorectal, gastric, liver, pancreatic, and uterine cancer [41]. Moreover, primary gastrointestinal and lung carcinomas, as well as metastatic bone disease, ovarian carcinoma, and brain tumors are often associated with high and very high morphine dosages [76].

#### Psychological factors

Patients with anxiety or sleep disturbance had a worse response (direct association). Psychological distress is often assessed by patients themselves via several health-related quality of life tools and in particular the EORTC QLQ-C30 (emotional functioning scale), a test able to assess many psychological parameters, including major depression, anxiety, or hostility that can make treatment more difficult [75]. Untreated anxiety has a negative impact on the management of cancer pain [95]. Sleep disturbances can be generated by anxiety but they might also be independent, thus more detailed information on sleep quality through specific sleep questionnaires (e.g. Pittsburgh Sleep Quality Index) could add further information [51].

#### Pain characteristics

Patients with neuropathic or breakthrough pain had a worse response (direct association). Breakthrough pain, including incidental pain, is a transient exacerbation of pain that occurs either spontaneously or in relation to a specific predictable or unpredictable trigger [96]. Neuropathic pain is defined as the pain caused by a lesion in the peripheral or central nervous system resulting from cancer or other causes such as chemotherapy. Despite significant progress in cancer research, few data are available yet on the pathophysiology of neuropathic pain due to cancer. The management of neuropathic pain is often inadequate and analgesic therapies need to be supported by adjuvants, such as anticonvulsants drugs, corticosteroids and antidepressants [97].

#### Other drugs

No clear association was found between the use of other drugs and opioid non-response. The use of nonsteroidal anti-inflammatory drugs (NSAIDs) and steroids is recommended in combination with weak or low dose opioids. Therefore, their usage may be directly linked to non-response because of poor treatment. On the other hand, proton pump inhibitors and laxatives might be prescribed to relieve adverse effects induced by high dose opioids. The use of these drugs may enhance response when concomitantly prescribed with high dose or strong opioids.

While some preliminary data were available, the relationship between non-response and cancer site, presence and location of metastases, and cognition was not defined.

In light of these results, we strongly suggest that future studies on cancer pain be designed with the specific aim of enhancing our understanding of opioid non-response and safety. Further information could be obtained through individual patient data meta-analysis, however it could be burdensome per se and problematic due to the low quality of original papers as well as the heterogeneity of the definition of “non-response” reported in the studies conducted up to now.

Moreover, the use of morphine dose to define drug response might be questionable. In our opinion, more efforts should be made to include proper treatment response evaluations, which can assess the real decrease in pain intensity through use of validated instruments, and not only through drug dose as a proxy. Further efforts should be made to precisely and routinely measure cancer pain in the strictest and most reliable manner available. This issue could be properly approached by clinicians according to Evidence Based Medicine parameters and not, as often still happens, according to their personal beliefs, hospital tradition or to unreliable self-reporting instruments.

### Strengths of this systematic review

- A comprehensive and robust systematic review in accordance with Cochrane Handbook and PRISMA guidelines.
- Search of two electronic database and assessment of the methodological quality of the included studies.
- All reviewing and data extraction was carried out by one author and double-checked by a second author; two other independent reviewers discussed and resolved any disagreement.
- Evaluation of a broad range of risk factors contributing to clinical response and adverse drug reactions to opioids.

### Limitations of the systematic review

- Definition of “non-response” reported in the studies included in the systematic review was heterogeneous.

### STOP pain project

In an effort to overcome the above mentioned problems, a longitudinal, nation-wide, paediatric study was planned by the Department of Neuroscience, Psychology, Drug Research and Children’s Health of the University of Florence, Italy and Anna Meyer Children’s University Hospital (Florence, Italy) entitled “STOP Pain - Suitable Treatment for Oncologic Paediatric Pain”.

In particular, the study uses more than one outcome to evaluate opioid responsiveness in terms of both opioid dosage and pain intensity assessment. This point raises the challenge of selecting a proper outcome measure of pain since it is a subjective experience that might be quite difficult to quantify [98]. In 2010, the AIRC (Italian Association for Cancer Research) financed this research program with the aim to evaluate the association between genetic factors and response to opioids in children. STOP Pain, comprehensive of the previous literature review, was a pilot study attempting to propose specific definition of clinical outcomes and their associated factors in a homogenous population (i.e. paediatric cancer-related pain patients). In fact, the main limitation of the study was the number of enrolled patients even if many of them were treated in a homogeneous way, i.e. titration of morphine by continuous infusion (60 out of 87).

Moreover, since children suffer from different types of cancer pain, the fact we did not characterize the nature of such pain (i.e. nociceptive, neuropathic, procedural, etc.) could represent another point of weakness of our study. Nevertheless, in some disease conditions, as well as in cancer, patients may have mixed pain consisting of somatic, visceral and neuropathic pain all at the same time or each separately at different times [99]. Clinical distinction between nociceptive and neuropathic pain is based on the anatomic origin of the stimulus, which was not clearly identifiable based on clinical data available for our pediatric patients.

In any case, planning of multicentric studies is pivotal to reach the appropriate sample size to address multiple comparison problems and capture minor genetic effects.

## Conclusions

It is our hope that the design of larger studies will consider the factors highlighted in the present work to enhance the understanding of opioid non-response and safety. And finally, we wish to underscore the necessity for studies on children in this field.

## Additional files

Additional file 1: BMC Cancer.doc, Full Search Strategy. (DOCX 17 kb)

Additional file 2: BMC Cancer.doc, Characteristics of the 74 studies included in the review. (RTF 506 kb)

Additional file 3: BMC Cancer.doc, Criteria for the quality assessment of the included studies in the review. (DOCX 62 kb)

Additional file 4: BMC Cancer.doc, The assessment of methodological quality for the included studies in the review. (DOC 174 kb)

Additional file 5: BMC Cancer.doc, PRISMA Checklist for the current review. (DOC 58 kb)
