# Supplementary material for: A systematic review of the risk factors for clinical response to opioids for all-age patients with cancer-related pain and presentation of the paediatric STOP pain study
Source: BMC Cancer. 2018 May 18;18:568. doi: 10.1186/s12885-018-4478-3 (PMC5960169; doi:10.1186/s12885-018-4478-3)
Supplement: Supplementary file 1 — BMC Cancer.doc, Full Search Strategy. (DOCX 17 kb) [file 12885_2018_4478_MOESM1_ESM.docx]

**Supplementary Table 1.** Full Search Strategy

**1a**. PUBMED search strategy

| [#6](https://www.ncbi.nlm.nih.gov/pubmed/advanced) | [Add](https://www.ncbi.nlm.nih.gov/pubmed/advanced) | Search (((#4 AND #1))) NOT Letter[Pt] AND Humans[Mesh] AND ( English[lang] OR Italian[lang])) |
| --- | --- | --- |
| [#](https://www.ncbi.nlm.nih.gov/pubmed/advanced)5 | [Add](https://www.ncbi.nlm.nih.gov/pubmed/advanced) | Search (#4 AND #1) |
| [#](https://www.ncbi.nlm.nih.gov/pubmed/advanced)4 | [Add](https://www.ncbi.nlm.nih.gov/pubmed/advanced) | Search (#2 OR #3) |
| [#](https://www.ncbi.nlm.nih.gov/pubmed/advanced)3 | [Add](https://www.ncbi.nlm.nih.gov/pubmed/advanced) | Search “cohort studies”[Mesh] OR “epidemiologic methods”[Mesh] OR “case-control studies”[Mesh] OR cohort*[TIAB] OR (case*[TIAB] AND control*[TIAB]) OR (case*[TIAB] AND series[TIAB]) |
| [#2](https://www.ncbi.nlm.nih.gov/pubmed/advanced) | [Add](https://www.ncbi.nlm.nih.gov/pubmed/advanced) | Search (“Random Allocation”[Mesh] OR “Double Blind Method”[Mesh] OR “Single Blind Method”[Mesh] OR “Clinical Trials as topic”[Mesh] OR “Cross-Over Studies”[Mesh] OR “Prospective Studies”[Mesh] OR “Placebos”[Mesh] OR ((clinical[TIAB] OR control[TIAB] OR controlled[TIAB]) AND (study[TIAB] OR trial[TIAB])) OR ((single[TIAB] OR double[TIAB] OR triple[TIAB]) AND (blind*[TIAB] OR mask*[TIAB])) OR ((randomised[TIAB] OR randomized[TIAB] OR random*[TIAB]) AND (assign*[TIAB] OR allocat*[TIAB] OR group[TIAB] OR grouped[TIAB] OR patients[TIAB] OR study[TIAB] OR trial[TIAB] OR distribut*[TIAB])) OR (crossover[TIAB] AND (design[TIAB] OR study[TIAB] OR trial[TIAB])) OR placebo[TIAB] OR placebos[TIAB] ) |
| #1 | [Add](https://www.ncbi.nlm.nih.gov/pubmed/advanced) | Search ((opioid[tiab] or opioids[tiab] or opiate[tiab] or opiates[tiab] or morphine[tiab] or fentanyl[tiab] or oxycodone[tiab] or methadone[tiab] or codeine[tiab] or OTFC[tiab] or sufentanil[tiab] or alfentanil[tiab] or remifentanil[tiab] or pethidine[tiab] or tramadol[tiab] or buprenorphine[tiab] or Opiate Alkaloids[Mesh]) AND (cancer*[tiab] or neoplasm*[tiab] or tumor[tiab] or tumors[tiab] or tumour[tiab] or tumours[tiab] or Neoplasms [MeSH])) |

**1b.** EMBASE search strategy

| #7 | #5 NOT #6 |
| --- | --- |
| #6 | [letter]/lim |
| #5 | #3 AND #4 AND ([english]/lim OR [italian]/lim) AND [humans]/lim |
| #4 | #1OR #2 |
| #3 | 'opiate'/exp OR opioid:ti,ab OR opioids:ti,ab OR opiate:ti,ab OR opiates:ti,ab OR morphine:ti,ab OR fentanyl:ti,ab OR oxycodone:ti,ab OR methadone:ti,ab OR codeine:ti,ab OR otfc:ti,ab OR sufentanil:ti,ab OR alfentanil:ti,ab OR remifentanil:ti,ab OR pethidine:ti,ab OR tramadol:ti,ab OR buprenorphine:ti,ab AND ('neoplasm'/exp OR cancer*:ti,ab OR neoplasm*:ti,ab OR tumor*:ti,ab OR tumour*:ti,ab) |
| #2 | 'cohort analysis'/exp OR 'epidemiology'/exp OR 'case control study'/exp OR cohort*:ti,ab OR (case*:ti,ab AND control*:ti,ab) OR (case*:ti,ab AND series:ti,ab) |
| #1 | 'randomization'/exp OR 'double blind procedure'/exp OR 'single blind procedure'/exp OR 'clinical trial (topic)'/exp OR 'crossover procedure'/exp OR 'prospective study'/exp OR 'placebo'/exp OR 'clinical trial'/exp OR (clinical:ti,ab OR control:ti,ab OR controlled:ti,ab AND (study:ti,ab OR trial:ti,ab)) OR (single:ti,ab OR double:ti,ab OR triple:ti,ab AND (blind*:ti,ab OR mask*:ti,ab)) OR (randomised:ti,ab OR randomized:ti,ab OR random*:ti,ab AND (assign*:ti,ab OR allocat*:ti,ab OR group:ti,ab OR grouped:ti,ab OR patients:ti,ab OR study:ti,ab OR trial:ti,ab OR distribut*:ti,ab)) OR (crossover:ti,ab AND (design:ti,ab OR study:ti,ab OR trial:ti,ab)) OR placebo:ti,ab OR placebos:ti,ab |
