# Supplementary material for: A systematic review of the risk factors for clinical response to opioids for all-age patients with cancer-related pain and presentation of the paediatric STOP pain study
Source: BMC Cancer. 2018 May 18;18:568. doi: 10.1186/s12885-018-4478-3 (PMC5960169; doi:10.1186/s12885-018-4478-3)
Supplement: Supplementary file 2 — BMC Cancer.doc, Characteristics of the 74 studies included in the review. (RTF 506 kb) [file 12885_2018_4478_MOESM2_ESM.rtf]

Supplementary Table 2. Characteristics of the 74 studies included in the review.

Study name	Study type	Location	Sample size	Age (mean±SD)	Gender (%female)	Cancer site	Other information	Drug	Definition of (NON)-Response
	
Anghelescu, 2015a	Retrospective chart review of the last 2 weeks of life of paediatric cancer patients treated with PCA (Patient Controlled Analgesia)	USA	44	13.6±6.6
(at death)	36	59% leukaemia/lymphoma; 41% solid tumor/brain tumor		O (ME)	High dose	
Anghelescu, 2015b	Retrospective study on 45 cancer patients receiving outpatient PCA over 54 months	USA	45	13.0±6.34	42.2	60% solid tumor; 8.9% brain tumor; 14% leukaemia and lymphoma		O (ME)	Discontinuation of PCA and high dose	
Appelgren, 1997	Retrospective study on 201 cancer patients who received intrathecal pain treatment	Europe (Sweden)	201	median age 61 (range: 18-91)	52.7	Multiple	Pain by surgery, radiation, chemotherapy and hormone therapy	O (ME)	High dose	
Arthur, 2015	Retrospective study of medical charts on 100 cancer patients (91 eligible) between February 2008 and March 2010 at the outpatient supportive care clinic in a comprehensive cancer center	USA	91	median age 58 (range: 29-90)	55.0	22.0% gastrointestinal; 20.9% head and neck; 11.0% lung; 9.9% breast; 8.8% gynaecologic; 7.7% genitourinary; 6.6% leukemia/lymphoma; 13.2% others	Ethnicity: 70.3% Caucasian

Cancer stage: 79.1% metastatic/refractory; 20.9% locally advanced
	O (ME)	High dose; opioid rotation	
Bercovitch, 1999	Retrospective medical records review of all 651 inpatients hospitalized between January 1996 and December 1997. The study included 453 patients who received morphine as first-line treatment on stable dosing ("around-the-clock dosing") for pain relief 	Asia 
(Israel)	453	 	40.6	32.0% gastrointestinal, 18.8% breast, 14.3% genitourinary, 14.1% lung, 20.8% others	73.3% white	M	High dose	
Bercovitch, 2004	Retrospective study of medical charts on 661 cancer patients treated by a home-care hospice (435 morphine-treated and 226 non-morphine-treated patients)	Asia 
(Israel)	661	62,5±13,2 	51.9	22.8% stomach, 10.3% colon, 0.9% esophagus, 15.0% breast, 15.9% lung, 5.1% prostate, 3.8% ear/nose/throat, 26.2% others	place of death: 62,6% home, 32,1% hospice, 5,3% other.	M 	High dose	
Bercovitch, 2006	Retrospective chart analysis on 97 terminal cancer patients	Asia 
(Israel)	97	73,3±12,8 	54.6	 	 	Oxycodone	High dose	
Bergman, 2015	Prospective observational study in 100 palliative cancer patients	Europe (Sweden)	100	median age 71 (range: 17-93)	43.0	20 gastrointestinal; 19 lung; 13 gynaecological; 13 pancreatic; 10 breast; 7 prostate; 5 hematologic; 3 head and neck; 3 malignant melanoma; 2 cholangiocarcinoma; 2 brain tumor; 3 others		Fentanyl	High dose	
Bruera, 1995	Prospective study including 277 patients with cancer pain	North America (Canada)	277	59±14.22  	58.8	23.1% genitourinary, 19.5% lung, 18.4% gastrointestinal, 17.3% breast, 10.5% head and neck, and 11.2% others	 	O (ME)	Poor pain control 	
Candrilli, 2009	Retrospective analysis of insurance claim data on 8836 opioid initiators with cancer (821 experienced constipation)	USA	8836	52.1 	57.6	 	 	O	ADR	
Cherny, 1994	Combined analysis of 4 controlled single-dose studies on 168 patients	USA	168	median age 61 (range: 18-91)	52.4	15.5% lung, 14.9% breast, 5.9% unknown,4.8% sarcoma, 4.8% prostate, 4.8% bladder, 4.8% esophagus	 	O (ME)	Low pain relief	
Chow, 2001	Survey with CAGE questionnaire in an outpatients palliative radiotherapy clinic in 128 patients	North America (Canada)	128	median age in patients without drinking problems 69 (range: 39-93);	46,9	34.4% lung, 20% breast, 14.8% prostate, 9.4% gastrointestinal, 13.3% unknown 	100% metastasis	O (ME)	High dose	
Collin, 1993 	Study on 44 patients who required oral morphine therapy for nociceptive pain over an 18-month period. After exclusion of 15 patients, study included 29 patients	Europe (France)	29	55.6±2.6 	62.1	20.7% lung, 20.7% gynaecological, 17.2% gastrointestinal, 13.8% melanoma, 10.3% head and neck, 10.3% breast, 7.0% urinary tract	 	M	High dose; high pain intensity  	
De Conno, 1996	Retrospective study on 196 advanced cancer outpatients	Europe (Italy)	196	median age 59 	48.3	33,2% head and neck; 14,8% lung, 12,2% breast	 	Methadone	Low Palliation Index	
Dougherty, 2003	Review of medical records (case series study) of 20 terminal cancer children 3 days before death	USA	20	9.2±6.3 	 	 	 	M	High dose	
Droney, 2008	Part of an observational study examining determinants of the response to oral morphine including 274 cancer patients	Europe (UK) 	274	 	 	 	 	M	ADR	
Edrington, 2004	Data from a randomized clinical trial (PROSELF©: PAIN CONTROL PROGRAM) 	USA	187	men 64.8±11.3;
women 57.5±12.0	69.52	-Men: 42.1% prostate; 26.3% lung; 3.5% colon; 28.1% others
-Women: 73.6% breast; 7.8% lung; 0.8% colon; 17.8% others	Around-the-clock (ATC) and/or as needed (PRN) analgesic prescription	O (ME)	High dose	
Faisinger, 2005	Study on 746 consecutive cancer patients. The study included 619 subjects with pain syndrome	North America (Canada)	619	66.0±13 	52.7	28.8% gastrointestinal, 24.4% lung, 19.5% genitourinary, 10.5% breast, 16.8% others	 	O (ME)	Long time to achieve stable pain control; high dose	
Faisinger, 2010	Study on 1100 consecutive cancer patients. The study included 944 subjects with pain syndrome	North America (Canada)	944	61.0±15	50.0	24.0% gastrointestinal, 22.0% lung, 15.0% genitourinary, 13.0% breast, 6.0% head and neck, 5.0%haematological, 4.0% unknown, 6.0% others	 	O (ME)	Long time to achieve stable pain control; high dose	
Fallon, 1999	Observational study on 50 advanced cancer patients with a prognosis of at least 4 weeks	Europe (UK) 	50	23-84 	52.0	 	 	M	 	
Flogegard, 2003	Prospective analysis of charts for morphine infusions in 72 patients referred to a paediatric oncology and haematology unit	Europe (Sweden)	72	median age 6,2 (range: 0,25-17,0)	56.0	32.0% ALL, 8.0% neuroblastoma, 7.0% soft tissue sarcoma, 7.0% AML; 7.0% Hodgkin, 7.0% non-Hodgkin	Aetiology of pain: 39% minor surgery, 33% major surgery, 17% tumour, 11% side effects; 	M 	High BTcP episode number	
Gagnon, 2000	Pilot prospective cohort study on 94 terminally ill cancer patients with a life expectancy shorter than two months. After the exclusion of 5 patients due to comatose status, 89 patients were enrolled	Canada	89	mean (median) 66.4 (68.0)	51.7	Primary cancer site:
32.6 respiratory tract;
30.3 digestive tract; 13.5 urinary tract; 23.6 others		O (ME)	High dose	
Glare, 1993	Retrospective, open, dose-ranging study on 24 patients with advanced cancer	USA	24	median age 64 (range: 26-81) 	50.0	20,8% lung, 16,7% prostate, 12,5% hematologic, 12,5% renal, 37.5% others	 	Oxycodone 	High dose	
Glare, 2006	Prospective survey on 56 advanced cancer patients	USA	42	median age 64 (range: 39-78) 	45.2	25% lung, 17% breast, 13% prostate, 13% colorectal; 	70% metastatic disease.	M (ME)	High dose	
Goksu, 2014	Prospective study on 100 patients with cancer pain	Turkey	100	median age 56	37	lung 30%, gastrointestinal 28%, breast 9%,
gynaecologic 9%	89% metastatic disease.	O (ME)	Persistent Pain-Free Survival (PPFS); VAS score; high dose	
Greco, 2011	Multicentre, prospective, longitudinal study on 1801 patients with cancer and pain	Europe (Italy)	1801	40,3% had BTcP at baseline; most patients are men; 	 	common primary tumor: lung, breast, colorectal; 	bone metastasis from 42,5% to 52,4%; neuropathic pain from 19,8% to 39,9%	O (ME)	High dose; switching	
Gretton, 2013	prospective observational study of 228 cancer patients taking oral preparations of morphine for moderate-to-severe cancer pain	Europe (UK) 	228
(212 had samples available for metabolite analysis)	57±14	52	breast 20%, gynaecological 8%, haematological 4%, head and neck 8%, lower gastrointestinal tract 6%, lung 12%, sarcoma 15%, upper gastrointestinal tract 8%, urogenital 10%, others 6%		M	Switching from morphine; morphine, M3G and M6G molar plasma concentration	
Hagen, 2007	Re-analysed data from three clinical trials on oral transmucosal Fentanyl citrate as a treatment for breakthrough pain including 257 cancer patients. From the original group, 188 were successfully treated with OTCF	North America (Canada)	188	median age 54.8 (range: 26-91)	56.0	23.0% breast, 19.0% lung, 12.0% gynaecologic, 10.0% colon-rectal, 8.2% lymphohematologic, 27.8% others	 	Fentanyl	High dose	
Hall, 2003	Retrospective review of 7201 (out of 15944) pharmacy records of patients with cancer diagnosis and who were prescribed transdermal fentanyl, sustained-release oral morphine, or oxycodone	USA	7201	70.0	49.6	31.0% lung, 11.7% colorectal, 8.1% breast, 7.4% carcinoma NS, 7.3% pancreatic, 6.4% prostatic, 28.1% others	 	M, Oxycodone, Fentanyl (ME)	High dose	
Hayashi, 2014	Retrospective study on cancer patients	Asia (Japan)	68	68.3±9.4	35.3	Lung 19.1%, stomach 1.2%, pancreas 11.7%, pharynx/parotid gland 10.3%, lips/tongue/chin 7.3%, gallbladder/bile duct 5.8%, rectum 5.8%, liver 4.11%, ureter/bladder 4.11%, uterus/ovary, kidney and breast 2.9%, bone marrow, small intestine, esophagus, prostate, skin, and others 1.5%		O (ME)	Opioid rotation; effective analgesia	
Hwang, 2002	Prospective, longitudinal study on 74 patients with poorly controlled cancer pain (patients with worst pain ≥4 out of 10) 	USA	74	median age 63 (range: 40-82)	 	32.0% lung, 22.0% prostate, 12.0% head and neck, 8.0% colorectal, 4.0% lymphoma, 22.0% other. 	median education level 12th grade; median number of pain sites 1 (1-5); pain diagnosis: 67% nociceptive pain, 34% bone pain, 34% neuropathic pain, 70% breakthrough pain, 40% mixed pain; 	O (ME)	Pain relief ≤ 80% 	
Kaiko, 1983	Reanalysis of data from clinical trial  on analgesics on 715 cancer patients with chronic pain who received injection of morphine sulphate	USA	715	 	59	 	87% white, 11% black, 1.5% oriental;	M 	High dose	
Kanbayashi, 2011	Retrospective review of care records of 76 hospitalised cancer patients who underwent opioid switching from oxycodone or morphine-sustained release tablet to transdermal fentanyl	Asia (Japan)	76	62.9±14.6	44.7	14.5% lung, 13.2% gastric, 6.6% myeloma, 6.6% breast, 5.3% colon, 5.3% pancreas, 5.3% ovarian, 5.3% pharyngeal, 37.9% others	 	Fentanyl	High dose	
Karavelis, 1996	Analysis of 90 patients with medically intractable cancer pain undergoing intraventricular morphine administrations	Europe (Greece)	90	median age 58 (range: 23-80) 	34.0	23% digestive system, 19% lung, 10% genitourinary, 9% breast, 4% head and neck, 2% miscellaneous, 33% others	 	M	Low pain relief	
Knudsen, 2011	International, multicentre, cross-sectional study of 2278 cancer pain treated with opioids	Europe (Norway)	2278	62.2±12.3 (range:18-96)	47.6	23.0% gastrointestinal, 18.3% lung, 13.3% breast, 11.6% prostate, 7.6% gynaecological, 7.3% urological, 5.8% hematologic, 5.5% head and neck, 2.7 unknown, 4.9% others	 	O (ME)	Low pain relief	
Knudsen, 2012	GROUP1 Multicentre, observational longitudinal study: cross-sectional analysis on 1529 patients with advanced cancer using opioids at the day of inclusion	Europe	1529	63.8±22-92 	47.2	28.1% gastrointestinal, 22.4% lung, 16.6% breast, 7.8% prostate, 6.1% urological, 5.5% head and neck, 5.4% gynaecological	most frequent location of metastases: 47,6% bone, 41,5% lymph node, 28,2% lung, 27,7% liver, 15,7% abdominal	O (ME)	No pain relief	
	GROUP2 Multicentre, observational longitudinal study: longitudinal analysis on 352 patients with advanced cancer newly referred to palliative care		352	64.8±26-88	38.6	27.1% gastrointestinal, 27.6% lung, 9.1% breast, 6.3% prostate, 8.8% urological, 8.0% head and neck, 3.7% gynaecological	most frequent location of metastases: 44,6% bone, 38,4% lymph node, 24,2% lung, 22,4% liver, 12,2% abdominal	O (ME)	No pain relief	
Korzeniewska-Eksterowicz, 2014	Retrospective analysis of medical records of 42 children with cancer treated at home, 21 of them started Palliative Sedation PS	Europe
(Poland)	42
(21 started PS)			For patients with PS:
solid tumor 26.2%, brain tumor 11.9%, bone tumor 9.5%, leukaemia 2.4%		M	high dose	
Kurita, 2008	Pilot study of 26 cancer patients (14 with opioids, OG) admitted to oncologic clinics	South America (Brazil)	26	52.4±12.9 	50.0	35.7% thorax,  21.4% abdomen, 7.1% members, 5.7% head/neck 	 	O	High pain intensity 	
Kurita, 2011	Prospective cross-sectional multicentre study on 1915 cancer patients with opioids for at least 3 days	Europe	1915	61.6	 	Most frequent cancer diagnosis: 20.1% gastrointestinal, 16.5% lung, 13.8% breast, 11.2% prostate;	Mini Mental State Examination (MMSE) mean score 29,5	O (ME)	High dose	
Kurita, 2015	Cross-sectional multicentre study (EPOS study) of 1147 patients treated for at least 3 days with one of the most frequently reported opioids (581 morphine; 298 oxycodone; 268 fentanyl)	Europe	1147	18-39yrs 5%; 
40-49yrs 12%;
50-59yrs 22%;
60-69yrs 31%;
70-79yrs 23%;
>80yrs 7%	48.7	Most frequent cancer diagnosis: 21% gastrointestinal; 15% breast; 15% lung; 12% prostate; 8% head and neck; 8% female reproductive organs; 7% urologic	Kidney disease 4%	M; Fentanyl;
oxycodone	ADR	
Li, 2010	Retrospective review of 150 cancer patients who received palliative cares in Taiwan between July 2005 and August 2008 during their second-last and last week of life	Asia (Taiwan)	150	60.3±14.1	52.7	11.3% head and neck; 12.0% respiratory; 50.7% gastrointestinal and hepatobiliary system; 8.7% breast; 11.3% urogenital  system; 0.7% hematopoietic-lymphatic system; 2.7% skin, sarcoma, connective tissue; 2.7% others	Opioids used: fentanyl, morphine and Demerol	O (ME)	High dose	
Liang, 2008	Cross-sectional study on 92 cancer outpatients of two teaching hospital	Asia (Taiwan)	92	56.4±12.2 (range: 30-92)  	41.3	35.9% head and neck, 18.5% colon/rectum, 14.1 breast, 31.5% others;	66.3% metastatic disease; pain>=3; 	O	Poor pain relief	
Liang, 2013	Descriptive and comparative study on 92 cancer patients who had been prescribed opioid analgesics for cancer-related pain	Asia (Taiwan)	92		41.3	Males:
-head/neck cancer 50%
-others 50%
Females:
-head/neck cancer 15.8%
-others 84.2%	61.1% metastatic disease	O (ME)	High opioid consumption; high average pain level	
Lin, 2011	Retrospective chart review of 94 patients who died of primary head and neck cancer admitted to a palliative care unit during a period of 2and 1/2 years	Asia (Taiwan)	94	54.3±12.5 	7.4	100.0% head and neck	87.2% metastatic disease; pain≥3; 	O (ME)	High dose	
Makimura, 2011	Prospective study on (50 ) 44 cancer patients treated with morphine (July 2009-March 2011)	Asia (Japan)	44	Range: 40-85  	50.0	30% others, 18% CRC, 9% gastric, 3% lung	 	M	High dose; persistence of pain 	
Mercadante, 1992	During a period of 2 months, 130 patients were followed at home until death using only a pharmacological approach to control pain according to the WHO ladders. After exclusion of 32 patients, study included 89 subjects	Europe (Italy)	89	60±7	51.0	36.6% lung, 21.4% breast, 14.3% genitourinary, 13.3% gastrointestinal, 9.2% head and neck, 13.3% others	 	O	Low pain relief	
Mercadante, 1997	Prospective longitudinal survey (one year) of 325 cancer patients [Italy]; 67 completed the study	Europe (Italy)	67	57% >65 years old	 43.0	Cancer diagnosis: 30% lung, 19% gastrointestinal, 16% urogenital, 10% breast, 7% liver/pancreas, 7% head/neck, 11% others	 	Dextropropoxyphene, M	High OEI %; high OEI mg	
Mercadante, 1998	Prospective study (Jan 1994-Sept 1997) of 107 head/neck cancer patients: 37 selected patients who, before death, required opioid therapy for more than 6 weeks	Europe (Italy)	107	median age 61.0	43.0	100.0 head and neck	 	O	High OEI %; high OEI mg	
Mercadante, 1999	Prospective study on 45 cancer patients followed at home	Europe (Italy)	45	60.6±11.9	53.0	24% lung,  15.5% breast, 13% colon/rectum, 8.5% ovary, 6.5% genitourinary, 6.5% pancreas, 24% others	 	Methadone	High MEI %; high MEI mg	
Mercadante, 2000a	181 patients who had been receiving opioid therapy for longer than 4 week with more than 18 years old within a study carried out in a sample of consecutive advance cancer patients who were followed at home	Europe (Italy)	181	65.9±11.3; (range: 31-90)	62.0	24.9% lung,  11.0% colon-rectum, 7.7% breast, 7.7% urinary, 7.7% head and neck, 6.6% prostate, 7.2% unknown, 27.7% other	 	Dextropropoxyphene, M (ME)	High OEI % 	
Mercadante, 2000b	Prospective study on 181 cancer patients followed at home	Europe (Italy)	181	 	 	24.9% lung, 10.5% colorectal, 7.7% breast, 7.7% head and neck, 7.7% urogenital, 6.6% prostate, 5% stomach,  29.9% others	 	Dextropropoxyphene, M	High OEI %; high OEI mg; high dose	
Mercadante, 2006	Prospective study on 100 cancer patients admitted to acute palliative care unit	Europe (Italy)	100	47% >65 years old	 	 	 	O (ME)	High OEI %; high OEI mg	
Mercadante, 2009	Prospective study carried out in a sample of 345 consecutive patients admitted to an acute and palliative care unit over a period of 2 years	Europe (Italy)	345	 	 	 	 	O (ME)	Switching	
Mercadante, 2011a	Survey (8 months) of 308 (170 assessed patients) cancer patients admitted to acute pain relief and palliative care unit	Europe (Italy)	308	65.1±12.2 	37.6	 	 	O	ADR	
Mercadante, 2011b	Retrospective study on 212 cancer patients in a period of 3 years admitted to acute pain relief and palliative care unit	Europe (Italy)	212	62.4±13.2 	44.3	25.5% gastrointestinal,  24.5% lung,  21.7% urogenital, 12.7% breast, 15.6% others	 	Oxycodone	High dose	
Mercadante, 2012	Retrospective records review of 345 (267 analysed patients) cancer patients undergoing opioid switching to methadone admitted to a palliative care unit during a period of 10 years	Europe (Italy)	267	62.5 (range: 34-82) 	42.6	24.3% lung , 18.3% gastrointestinal,  13.9% urogenital, 11.3% breast, 11% pancreas, 10.1% liver, 11.0% others	 	Methadone	High dose	
Miura, 2014	Retrospective charts review of 158 patients who died in palliative care unit.	Asia
(Japan)	158	Median 64 (range: 30-89)	43.7			O (ME)	High dose	
Morita, 1999	GROUP1 Cross-sectional study on 150 terminally-ill cancer patients admitted to hospice from Sept 1996 to Aug 1997 	Asia (Japan)	150	67±13	43.0	51.0% lung/pleura, 32.0% bone, 27.0% peritoneum, 26.0% liver/biliary system, 19.0% stomach/pancreas, 17.0%colon/rectum, 13.0% brain	 	O (ME)	High dose	
	GROUP2 Cohort study on 200 terminally-ill cancer patients admitted to hospice from Apr 1997 to Aug 1998		200	65±13	43.0	44.0% lung/pleura, 44.0% liver/biliary, 41.0% peritoneum, 32.0% bone, 23.0% stomach/pancreas, 16.0%colon/rectum, 15.0% brain	 	O (ME)	High dose	
Naito, 2012	Prospective study of 47 cancer patients receiving oxycodone as a starting opioid	Asia (Japan)	47	66 (range: 60-74)	29.8	21.3% pharyngeal, 17.0% lung, 8.5% multiple myeloma, 6.3% gastric,  6.4% colorectal, 4.3% pancreas, 4.3% prostate, 31.9% others	 	Oxycodone	High OEI % 	
Novy, 2012	Retrospective records review of 522 (final: 146 non-smokers and 46 smokers) cancer patients  admitted to a pain management service during a period of 6 months	USA	192	54.8±13.7 	51.8	18.5% gastrointestinal, 15.0% haematological, 14.6% head/neck, 11.5% breast, 11.3% lung, 6.8% skin, 6.4% urogenital, 6.4% gynaecological, 9.5% others	 	O (ME)	High dose	
Ozalp, 2003	Prospective study on 99 patients with breast cancer undergoing radical mastectomy. Relationship between preoperative anxiety and depression, and pain intensity, opioid consumption and degree of dissatisfaction	Turkey	99	43±9	100	100% breast cancer		O	Total opioid dose	
Parsons, 2008	Retrospective charts review of 665 (final: 598) cancer patients seen at the Palliative Care Clinic before Jan 2007 to find first 100 consecutive CAGE+ (four-item screening survey for alcoholism) patients	USA	598	CAGE-/CAGE+                      60 (16-91)/ 58 (28-87)  	53.0 / 32.0	23.0%/23.0% gastrointestinal, 22.0%/19.0% lung, 12.0%/8.0% urologic, 9.0%/24.0% head/neck, 9.0%/5.0% breast, 8.0%/6.0% gynaecologic, 3.0%/3.0%, 13.0%/12.0% others;	race: 75.0%/74.0% white	O (ME)	High dose	
Pina, 2015	Cross-sectional and related longitudinal study on 371 consecutive cancer patients	Europe
(Portugal)	371	62.1±14.3	53.6	24.8% head and neck;
2.7% lung; 22.1% gastrointestinal; 11.6 %breast; 21.3% genitourinary; 17.5% others	70.9% metastatic disease

Cancer pain characteristics:
-Nociceptive visceral 30.2%
-Nociceptive soft tissue 58.8%
-Nociceptive bone pain 40.2% 
-Neuropathic pain 43.4%
-Neuropathic pain or clinically mixed 77.4%
-Episodic incident 59.3%
-Episodic breakthrough 42.9%	O (ME)	High dose	
Radha Krishna, 2010	Retrospective review of case notes of 238 terminal cancer patients who had passed in a 95-bedded oncology ward between September 2006 and September 2007	Singapore	238	Median (range) 62 (15-96)	55.5%	17.6% lung; 16.0% colon; 10.5% breast; 8.0% hepatocellular; 6.3% unknown; 5.5% ENT; 5.0% stomach; 2.9% ovary; 2.1% prostate; 2.1% pancreas; 1.7% nasopharyngeal; 1.3% oesophagus; 21.0% others		O (ME)	High dose	
Rees, 1990	Retrospective survey of hospice records of all 1383 patients who died from cancer in hospice during a period of 9 years	Europe (UK) 	1383	range: 19-96 	58.3	19.0% lung; 15.5% breast; 14.7% colon-rectum; 7.0% stomach	 	M, diamorphine (ME)	High dose	
Riley, 2006 	Prospective study on all patients with cancer pain who required treatment with oral morphine for pain control. The study included 186 subjects	Europe (UK) 	186	57.6± 12.9 	54.8	21.0% breast, 14.0% sarcoma, 10.7% lung, 9.7% head and neck, 8.6% gynaecological, 7.0% renal cell, 6.4% lower gastrointestinal tract, 5.9% haematological, 16.7% others 	87.1% caucasian 	M	Switching	
Ripamonti, 2009	159 enrolled. Uncontrolled phase 4 clinical trial (Oct 2003-Aug 2005) on cancer patients naive to strong opioids with pain score >= 5	Europe (Italy)	159	64.9±10.7	35.8	22.4% lung, 21.8% gastrointestinal ,  11.5% breast, 7.1% prostate, 5.8% pancreas, others 31.4%	 	M	High pain intensity; high OEI	
Salminen, 2013	Sub-cohort (EPOS study) of 22 mesothelioma and 88 lung cancer patients	Europe
(Finland)	110	63.0±9.6	23.0	 	 	O (ME)	High dose	
Stromgren, 2004	Prospective study on 267 (final 175) cancer patients admitted to a specialist palliative care unit during a period of 2 years	Europe (Denmark)	175	62.8 (range: 37-91) 	56.0	26.3% respiratory system, 20.6% gastrointestinal tract, 17.1% breast, 16.6% genitourinary, 6.9% gynaecologic, 4.6% head/neck, 8.0% others	 	O (ME)	Poor pain relief	
Syrjala, 1995	Study on 358 patients receiving their first bone marrow transplantation	USA	358	range 18-62 	45.3	 	94% Caucasians	O (ME)	Worst mouth pain 	
Takase, 2011	Prospective study on 43 (23 with mild pain) head/neck cancer patients admitted to hospital between Apr 2004 and Sep 2008	Asia (Japan)	43	63.6±2.2	8.7	23.7% ANL; 11.2% ALL; 38.3% CML; 14.8% non-Hodgkin lymphoma	 	Oxycodone	High dose	
Utsumi, 2015	Retrospective study on medical records of 189 patients with gynaecological malignancies	Asia
(Japan)	189	Median 58.3	100	Ovary 42.3%, cervix 53.0%, corpus 27.0%, vaginal/vulva 2.6%	64% recurrence	O (ME)	High dose	
Viganò, 1998	Retrospective records review of 197 cancer patients admitted to a palliative care unit	North America (Canada)	197	67.5±11.1	52.8	31.0% gastrointestinal; 22.0% lung; 22.0% genitourinary; 13.0% breast	 	O (ME)	High dose	
Zyczkowska, 2013	Retrospective observational study in terminally ill digestive tract cancer patients	Europe
(Poland)	344	20.7% under 60yrs; 36.3% between 60 and 74yrs; 43.0% above 74yrs	42.44	100% digestive tract		O (ME)	High dose	

Reference
1	Anghelescu DL, Snaman JM, Trujillo L, Sykes AD, Yuan Y, Baker JN: Patient-controlled analgesia at the end of life at a pediatric oncology institution. Pediatric blood & cancer 2015, 62(7):1237-1244.
2	Anghelescu DL, Zhang K, Faughnan LG, Pei D: The Safety and Effectiveness of Patient-controlled Analgesia in Outpatient Children and Young Adults With Cancer: A Retrospective Study. Journal of pediatric hematology/oncology 2015, 37(5):378-382.
3	Appelgren L, Nordborg C, Sjoberg M, Karlsson PA, Nitescu P, Curelaru I: Spinal epidural metastasis: implications for spinal analgesia to treat "refractory" cancer pain. Journal of pain and symptom management 1997, 13(1):25-42.
4	Arthur J, Yennurajalingam S, Nguyen L, Tanco K, Chisholm G, Hui D, Bruera E: The routine use of the Edmonton Classification System for Cancer Pain in an outpatient supportive care center. Palliative & supportive care 2015, 13(5):1185-1192.
5	Bercovitch M, Adunsky A: High dose controlled-release oxycodone in hospice care. Journal of pain & palliative care pharmacotherapy 2006, 20(4):33-39.
6	Bercovitch M, Adunsky A: Patterns of high-dose morphine use in a home-care hospice service: should we be afraid of it? Cancer 2004, 101(6):1473-1477.
7	Bercovitch M, Waller A, Adunsky A: High dose morphine use in the hospice setting. A database survey of patient characteristics and effect on life expectancy. Cancer 1999, 86(5):871-877.
8	Bergman P, Sperneder S, Hoijer J, Bergqvist J, Bjorkhem-Bergman L: Low vitamin D levels are associated with higher opioid dose in palliative cancer patients--results from an observational study in Sweden. PLoS One 2015, 10(5):e0128223.
9	Bruera E, Schoeller T, Wenk R, MacEachern T, Marcelino S, Hanson J, Suarez-Almazor M: A prospective multicenter assessment of the Edmonton staging system for cancer pain. Journal of pain and symptom management 1995, 10(5):348-355.
10	Candrilli SD, Davis KL, Iyer S: Impact of constipation on opioid use patterns, health care resource utilization, and costs in cancer patients on opioid therapy. Journal of pain & palliative care pharmacotherapy 2009, 23(3):231-241.
11	Cherny NI, Thaler HT, Friedlander-Klar H, Lapin J, Foley KM, Houde R, Portenoy RK: Opioid responsiveness of cancer pain syndromes caused by neuropathic or nociceptive mechanisms: a combined analysis of controlled, single-dose studies. Neurology 1994, 44(5):857-861.
12	Chow E, Connolly R, Wong R, Franssen E, Fung KW, Harth T, Pach B, Andersson L, Schueller T, Stefaniuk K et al: Use of the CAGE questionnaire for screening problem drinking in an out-patient palliative radiotherapy clinic. Journal of pain and symptom management 2001, 21(6):491-497.
13	Cohen MR, Pickar D, Dubois M, Bunney WE, Jr.: Stress-induced plasma beta-endorphin immunoreactivity may predict postoperative morphine usage. Psychiatry research 1982, 6(1):7-12.
14	Collin E, Poulain P, Gauvain-Piquard A, Petit G, Pichard-Leandri E: Is disease progression the major factor in morphine 'tolerance' in cancer pain treatment? Pain 1993, 55(3):319-326.
15	De Conno F, Groff L, Brunelli C, Zecca E, Ventafridda V, Ripamonti C: Clinical experience with oral methadone administration in the treatment of pain in 196 advanced cancer patients. Journal of clinical oncology : official journal of the American Society of Clinical Oncology 1996, 14(10):2836-2842.
16	Dougherty M, DeBaun MR: Rapid increase of morphine and benzodiazepine usage in the last three days of life in children with cancer is related to neuropathic pain. The Journal of pediatrics 2003, 142(4):373-376.
17	Droney J, Ross J, Gretton S, Welsh K, Sato H, Riley J: Constipation in cancer patients on morphine. Supportive care in cancer : official journal of the Multinational Association of Supportive Care in Cancer 2008, 16(5):453-459.
18	Edrington JM, Paul S, Dodd M, West C, Facione N, Tripathy D, Koo P, Schumacher K, Miaskowski C: No evidence for sex differences in the severity and treatment of cancer pain. Journal of pain and symptom management 2004, 28(3):225-232.
19	Fainsinger RL, Nekolaichuk C, Lawlor P, Hagen N, Bercovitch M, Fisch M, Galloway L, Kaye G, Landman W, Spruyt O et al: An international multicentre validation study of a pain classification system for cancer patients. European journal of cancer (Oxford, England : 1990) 2010, 46(16):2896-2904.
20	Fainsinger RL, Nekolaichuk CL, Lawlor PG, Neumann CM, Hanson J, Vigano A: A multicenter study of the revised Edmonton Staging System for classifying cancer pain in advanced cancer patients. Journal of pain and symptom management 2005, 29(3):224-237.
21	Fallon MT, Hanks GW: Morphine, constipation and performance status in advanced cancer patients. Palliative medicine 1999, 13(2):159-160.
22	Flogegard H, Ljungman G: Characteristics and adequacy of intravenous morphine infusions in children in a paediatric oncology setting. Medical and pediatric oncology 2003, 40(4):233-238.
23	Flynn BC, Nemergut EC: Postoperative nausea and vomiting and pain after transsphenoidal surgery: a review of 877 patients. Anesthesia and analgesia 2006, 103(1):162-167, table of contents.
24	Gagnon P, Allard P, Masse B, DeSerres M: Delirium in terminal cancer: a prospective study using daily screening, early diagnosis, and continuous monitoring. Journal of pain and symptom management 2000, 19(6):412-426.
25	Glare P, Walsh D, Sheehan D: The adverse effects of morphine: a prospective survey of common symptoms during repeated dosing for chronic cancer pain. The American journal of hospice & palliative care 2006, 23(3):229-235.
26	Glare PA, Walsh TD: Dose-ranging study of oxycodone for chronic pain in advanced cancer. Journal of clinical oncology : official journal of the American Society of Clinical Oncology 1993, 11(5):973-978.
27	Goksu SS, Bozcuk H, Uysal M, Ulukal E, Ay S, Karasu G, Soydas T, Coskun HS, Ozdogan M, Savas B: Determinants of opioid efficiency in cancer pain: a comprehensive multivariate analysis from a tertiary cancer centre. Asian Pacific journal of cancer prevention : APJCP 2014, 15(21):9301-9305.
28	Greco MT, Corli O, Montanari M, Deandrea S, Zagonel V, Apolone G: Epidemiology and pattern of care of breakthrough cancer pain in a longitudinal sample of cancer patients: results from the Cancer Pain Outcome Research Study Group. The Clinical journal of pain 2011, 27(1):9-18.
29	Hagen NA, Fisher K, Victorino C, Farrar JT: A titration strategy is needed to manage breakthrough cancer pain effectively: observations from data pooled from three clinical trials. Journal of palliative medicine 2007, 10(1):47-55.
30	Hall S, Gallagher RM, Gracely E, Knowlton C, Wescules D: The terminal cancer patient: effects of age, gender, and primary tumor site on opioid dose. Pain medicine (Malden, Mass) 2003, 4(2):125-134.
31	Hwang SS, Chang VT, Fairclough DL, Kasimis B: Development of a cancer pain prognostic scale. Journal of pain and symptom management 2002, 24(4):366-378.
32	Kaiko RF, Wallenstein SL, Rogers AG, Houde RW: Sources of variation in analgesic responses in cancer patients with chronic pain receiving morphine. Pain 1983, 15(2):191-200.
33	Kaiko RF: Age and morphine analgesia in cancer patients with postoperative pain. Clinical pharmacology and therapeutics 1980, 28(6):823-826.
34	Kanbayashi Y, Hosokawa T, Okamoto K, Fujimoto S, Konishi H, Otsuji E, Yoshikawa T, Takagi T, Miki T, Taniwaki M: Factors predicting requirement of high-dose transdermal fentanyl in opioid switching from oral morphine or oxycodone in patients with cancer pain. The Clinical journal of pain 2011, 27(8):664-667.
35	Karavelis A, Foroglou G, Selviaridis P, Fountzilas G: Intraventricular administration of morphine for control of intractable cancer pain in 90 patients. Neurosurgery 1996, 39(1):57-61; discussion 61-52.
36	Knudsen AK, Brunelli C, Kaasa S, Apolone G, Corli O, Montanari M, Fainsinger R, Aass N, Fayers P, Caraceni A et al: Which variables are associated with pain intensity and treatment response in advanced cancer patients?--Implications for a future classification system for cancer pain. European journal of pain (London, England) 2011, 15(3):320-327.
37	Knudsen AK, Brunelli C, Klepstad P, Aass N, Apolone G, Corli O, Montanari M, Caraceni A, Kaasa S: Which domains should be included in a cancer pain classification system? Analyses of longitudinal data. Pain 2012, 153(3):696-703.
38	Kurita GP, de Mattos Pimenta CA: Cognitive impairment in cancer pain patients receiving opioids: a pilot study. Cancer nursing 2008, 31(1):49-57.
39	Kurita GP, Lundstrom S, Sjogren P, Ekholm O, Christrup L, Davies A, Kaasa S, Klepstad P, Dale O: Renal function and symptoms/adverse effects in opioid-treated patients with cancer. Acta anaesthesiologica Scandinavica 2015, 59(8):1049-1059.
40	Kurita GP, Sjogren P, Ekholm O, Kaasa S, Loge JH, Poviloniene I, Klepstad P: Prevalence and predictors of cognitive dysfunction in opioid-treated patients with cancer: a multinational study. Journal of clinical oncology : official journal of the American Society of Clinical Oncology 2011, 29(10):1297-1303.
41	Li M-H YE-T, Huang S-C, Wang H-M, Su W-R, Lai Y-L: Clinical Experience With Strong Opioids in Pain Control of Terminally ill Cancer Patients in Palliative Care Settings in Taiwan. Journal of Experimental and Clinical Medicine 2010, 2(6):292-296.
42	Liang SY, Yates P, Edwards H, Tsay SL: Factors influencing opioid-taking self-efficacy and analgesic adherence in Taiwanese outpatients with cancer. Psycho-oncology 2008, 17(11):1100-1107.
43	Lin YL, Lin IC, Liou JC: Symptom patterns of patients with head and neck cancer in a palliative care unit. Journal of palliative medicine 2011, 14(5):556-559.
44	Makimura C, Arao T, Matsuoka H, Takeda M, Kiyota H, Tsurutani J, Fujita Y, Matsumoto K, Kimura H, Otsuka M et al: Prospective study evaluating the plasma concentrations of twenty-six cytokines and response to morphine treatment in cancer patients. Anticancer research 2011, 31(12):4561-4568.
45	Mercadante S, Casuccio A, Agnello A, Barresi L: Methadone response in advanced cancer patients with pain followed at home. Journal of pain and symptom management 1999, 18(3):188-192.
46	Mercadante S, Casuccio A, Pumo S, Fulfaro F: Factors influencing the opioid response in advanced cancer patients with pain followed at home: the effects of age and gender. Supportive care in cancer : official journal of the Multinational Association of Supportive Care in Cancer 2000, 8(2):123-130.
47	Mercadante S, Casuccio A, Pumo S, Fulfaro F: Opioid responsiveness-primary diagnosis relationship in advanced cancer patients followed at home. Journal of pain and symptom management 2000, 20(1):27-34.
48	Mercadante S, Dardanoni G, Salvaggio L, Armata MG, Agnello A: Monitoring of opioid therapy in advanced cancer pain patients. Journal of pain and symptom management 1997, 13(4):204-212.
49	Mercadante S, Ferrera P, Casuccio A: Prevalence of opioid-related dysuria in patients with advanced cancer having pain. The American journal of hospice & palliative care 2011, 28(1):27-30.
50	Mercadante S, Ferrera P, David F, Casuccio A: The use of high doses of oxycodone in an acute palliative care unit. The American journal of hospice & palliative care 2011, 28(4):242-244.
51	Mercadante S, Ferrera P, Villari P, Casuccio A, Intravaia G, Mangione S: Frequency, indications, outcomes, and predictive factors of opioid switching in an acute palliative care unit. Journal of pain and symptom management 2009, 37(4):632-641.
52	Mercadante S, Ferrera P, Villari P, Casuccio A: Opioid escalation in patients with cancer pain: the effect of age. Journal of pain and symptom management 2006, 32(5):413-419.
53	Mercadante S, Maddaloni S, Roccella S, Salvaggio L: Predictive factors in advanced cancer pain treated only by analgesics. Pain 1992, 50(2):151-155.
54	Mercadante S: Opioid responsiveness in patients with advanced head and neck cancer. Supportive care in cancer : official journal of the Multinational Association of Supportive Care in Cancer 1998, 6(5):482-485.
55	Mercadante S: Switching methadone: a 10-year experience of 345 patients in an acute palliative care unit. Pain medicine (Malden, Mass) 2012, 13(3):399-404.
56	Morita T, Tsunoda J, Inoue S, Chihara S: Contributing factors to physical symptoms in terminally-ill cancer patients. Journal of pain and symptom management 1999, 18(5):338-346.
57	Naito T, Tashiro M, Yamamoto K, Ohnishi K, Kagawa Y, Kawakami J: Impact of cachexia on pharmacokinetic disposition of and clinical responses to oxycodone in cancer patients. European journal of clinical pharmacology 2012, 68(10):1411-1418.
58	Novy DM, Lam C, Gritz ER, Hernandez M, Driver LC, Koyyalagunta D: Distinguishing features of cancer patients who smoke: pain, symptom burden, and risk for opioid misuse. The journal of pain : official journal of the American Pain Society 2012, 13(11):1058-1067.
59	Ozalp G, Sarioglu R, Tuncel G, Aslan K, Kadiogullari N: Preoperative emotional states in patients with breast cancer and postoperative pain. Acta anaesthesiologica Scandinavica 2003, 47(1):26-29.
60	Park JE, Kim KI, Yoon SS, Hahm BJ, Lee SM, Yoon JH, Shin WG, Lee HS, Oh JM: Psychological distress as a negative survival factor for patients with hematologic malignancies who underwent allogeneic hematopoietic stem cell transplantation. Pharmacotherapy 2010, 30(12):1239-1246.
61	Parsons HA, Delgado-Guay MO, El Osta B, Chacko R, Poulter V, Palmer JL, Bruera E: Alcoholism screening in patients with advanced cancer: impact on symptom burden and opioid use. Journal of palliative medicine 2008, 11(7):964-968.
62	Pickar D, Cohen MR, Dubois M: The relationship of plasma cortisol and beta-endorphin immunoreactivity to surgical stress and postoperative analgesic requirement. General hospital psychiatry 1983, 5(2):93-98.
63	Pina P, Sabri E, Lawlor PG: Characteristics and associations of pain intensity in patients referred to a specialist cancer pain clinic. Pain research & management : the journal of the Canadian Pain Society = journal de la societe canadienne pour le traitement de la douleur 2015, 20(5):249-254.
64	Radha Krishna LK, Poulose JV, Tan BS, Goh C: Opioid use amongst cancer patients at the end of life. Annals of the Academy of Medicine, Singapore 2010, 39(10):790-797.
65	Rees WD: Opioid needs of terminal care patients: variations with age and primary site. Clinical oncology (Royal College of Radiologists (Great Britain)) 1990, 2(2):79-83.
66	Riley J, Ross JR, Rutter D, Wells AU, Goller K, du Bois R, Welsh K: No pain relief from morphine? Individual variation in sensitivity to morphine and the need to switch to an alternative opioid in cancer patients. Supportive care in cancer : official journal of the Multinational Association of Supportive Care in Cancer 2006, 14(1):56-64.
67	Ripamonti CI, Campa T, Fagnoni E, Brunelli C, Luzzani M, Maltoni M, De Conno F: Normal-release oral morphine starting dose in cancer patients with pain. The Clinical journal of pain 2009, 25(5):386-390.
68	Salminen EK, Silvoniemi M, Syrjanen K, Kaasa S, Kloke M, Klepstad P: Opioids in pain management of mesothelioma and lung cancer patients. Acta oncologica (Stockholm, Sweden) 2013, 52(1):30-37.
69	Stromgren AS, Groenvold M, Petersen MA, Goldschmidt D, Pedersen L, Spile M, Irming-Pedersen G, Sjogren P: Pain characteristics and treatment outcome for advanced cancer patients during the first week of specialized palliative care. Journal of pain and symptom management 2004, 27(2):104-113.
70	Syrjala KL, Chapko ME: Evidence for a biopsychosocial model of cancer treatment-related pain. Pain 1995, 61(1):69-79.
71	Takase H, Sakata T, Yamano T, Sueta T, Nomoto S, Nakagawa T: Advantage of early induction of opioid to control pain induced by irradiation in head and neck cancer patients. Auris, nasus, larynx 2011, 38(4):495-500.
72	Vigano A, Bruera E, Suarez-Almazor ME: Age, pain intensity, and opioid dose in patients with advanced cancer. Cancer 1998, 83(6):1244-1250.
73	Yang JC, Clark WC, Tsui SL, Ng KF, Clark SB: Preoperative Multidimensional Affect and Pain Survey (MAPS) scores predict postcolectomy analgesia requirement. The Clinical journal of pain 2000, 16(4):314-320.
74	¯yczkowska J GT, Kleja J, Filipczak-Bryniarska I, Wrzosek A, Wordliczek J: Age influence on opioid consumption in terminally ill digestive cancer patients. Medycyna Paliatywna w Praktyce 2013, 7(2):50-54.
