# Supplementary material for: A systematic review of the risk factors for clinical response to opioids for all-age patients with cancer-related pain and presentation of the paediatric STOP pain study
Source: BMC Cancer. 2018 May 18;18:568. doi: 10.1186/s12885-018-4478-3 (PMC5960169; doi:10.1186/s12885-018-4478-3)
Supplement: Supplementary file 3 — BMC Cancer.doc, Criteria for the quality assessment of the included studies in the review. (DOCX 62 kb) [file 12885_2018_4478_MOESM3_ESM.docx]

**Supplementary Table 3.** Criteria for the quality assessment of the included studies in the review.


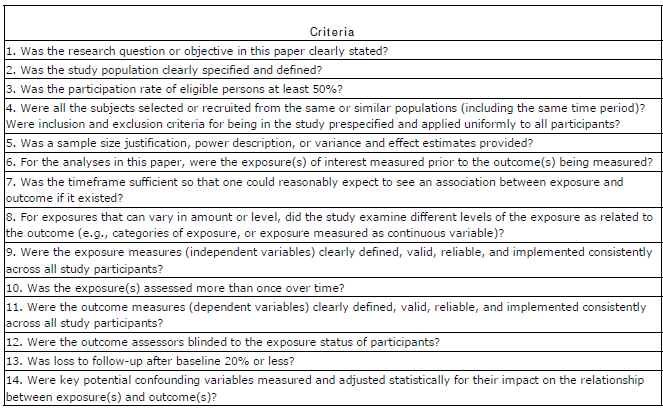


**Reference**

1. Quality Assessment Tool for Observational Cohort and Cross-Sectional Studies [http://www.nhlbi.nih.gov/health-pro/guidelines/in-develop/cardiovascular-risk-reduction/tools/cohort.htm]
