# Supplementary material for: A systematic review of the risk factors for clinical response to opioids for all-age patients with cancer-related pain and presentation of the paediatric STOP pain study
Source: BMC Cancer. 2018 May 18;18:568. doi: 10.1186/s12885-018-4478-3 (PMC5960169; doi:10.1186/s12885-018-4478-3)
Supplement: Supplementary file 4 — BMC Cancer.doc, The assessment of methodological quality for the included studies in the review. (DOC 174 kb) [file 12885_2018_4478_MOESM4_ESM.doc]

**Supplementary Table 4.** The assessment of methodological quality for the included studies in the review.

|  | Criteria | | | | | | | | | | | | | |
| --- | --- | --- | --- | --- | --- | --- | --- | --- | --- | --- | --- | --- | --- | --- |
| Study name | 1 | 2 | 3 | 4 | 5 | 6 | 7 | 8 | 9 | 10 | 11 | 12 | 13 | 14 |
| Anghelescu, 2015a | yes | yes | no | yes | NR | yes | yes | NA | yes | yes | yes | NA | yes | yes |
| Anghelescu, 2015b | yes | yes | NR | yes | NR | yes | yes | NA | yes | yes | yes | NA | yes | yes |
| Appelgren, 1997 | yes | no | NR | yes | NR | yes | yes | NA | yes | yes | yes | NA | yes | yes |
| Arthur, 2015 | yes | yes | yes | yes | NR | yes | yes | NA | yes | yes | yes | NA | yes | yes |
| Bercovitch, 1999 | yes | yes | yes | yes | NR | yes | yes | NA | yes | yes | yes | NA | yes | yes |
| Bercovitch, 2004 | yes | yes | yes | yes | NR | yes | yes | yes | yes | yes | yes | NA | yes | yes |
| Bercovitch, 2006 | yes | yes | NR | yes | NR | yes | yes | yes | yes | yes | yes | NA | yes | yes |
| Bergman, 2015 | yes | yes | NR | yes | NR | yes | yes | no | yes | yes | yes | NA | yes | yes |
| Bruera, 1995 | yes | no | NR | yes | NR | yes | yes | yes | yes | yes | yes | yes | yes | yes |
| Candrilli, 2009 | yes | yes | yes | yes | NR | yes | yes | NA | yes | yes | yes | NA | yes | yes |
| Cherny, 1994 | yes | yes | NR | yes | NR | yes | yes | NA | yes | yes | yes | yes | yes | yes |
| Chow, 2001 | yes | yes | NR | yes | NR | yes | yes | NA | yes | yes | yes | NA | yes | no |
| Collin, 1993 | yes | no | NR | yes | NR | yes | yes | NA | yes | yes | yes | yes | no | yes |
| De Conno, 1996 | yes | yes | NR | yes | NR | yes | yes | NA | yes | yes | yes | NA | yes | no |
| Dougherty, 2003 | yes | yes | no | yes | NR | yes | yes | NA | yes | yes | yes | NA | no | no |
| Droney, 2008 | yes | no | NR | yes | NR | yes | yes | yes | yes | yes | yes | NA | yes | NR |
| Edrington, 2004 | yes | yes | NR | yes | NR | yes | yes | NA | yes | yes | yes | NA | yes | yes |
| Faisinger, 2005 | yes | yes | NR | yes | NR | yes | yes | NA | yes | yes | yes | NA | yes | yes |
| Faisinger, 2010 | yes | yes | NR | yes | NR | yes | yes | NA | yes | yes | yes | NA | yes | yes |
| Fallon, 1999 | yes | no | NR | yes | NR | yes | yes | NA | yes | yes | yes | NA | NR | NR |
| Flogegard, 2003 | yes | yes | NR | yes | NR | yes | yes | NA | yes | yes | yes | NA | NR | yes |
| Gagnon, 2000 | yes | yes | yes | yes | NR | yes | yes | NA | yes | yes | yes | NA | yes | no |
| Glare, 1993 | yes | yes | NR | yes | NR | yes | yes | NA | yes | yes | yes | NA | yes | NR |
| Glare, 2006 | yes | no | NR | yes | NR | yes | yes | NA | yes | yes | yes | NA | yes | yes |
| Goksu, 2014 | yes | yes | NR | yes | NR | yes | yes | NA | yes | yes | yes | NA | yes | yes |
| Greco, 2011 | yes | yes | NR | yes | yes | yes | yes | NA | yes | yes | yes | NA | yes | yes |
| Gretton, 2013 | yes | no | NR | yes | NR | yes | yes | NA | yes | yes | yes | NA | yes | no |
| Hagen, 2007 | yes | no | NR | NA | NR | yes | NR | NA | yes | yes | yes | NA | yes | yes |
| Hall, 2003 | yes | yes | no | yes | NR | yes | no | yes | yes | yes | yes | NA | yes | yes |
| Hwang, 2002 | yes | yes | yes | yes | NR | yes | yes | NA | yes | yes | yes | NA | yes | yes |
| Hayashi, 2014 | yes | yes | NR | yes | NR | yes | yes | yes | yes | yes | yes | NA | yes | yes |
| Kaiko, 1983 | yes | no | NR | yes | NR | yes | yes | yes | yes | yes | yes | yes | yes | yes |
| Kanbayashi, 2011 | yes | yes | NR | yes | NR | yes | yes | NA | yes | yes | yes | NA | yes | yes |
| Karavelis, 1996 | yes | no | NR | yes | NR | yes | yes | NA | yes | yes | yes | NA | yes | yes |
| Knudsen, 2011 | yes | yes | yes | yes | NR | yes | yes | NA | yes | yes | yes | NA | yes | yes |
| Knudsen, 2012 | yes | yes | yes | yes | NR | yes | yes | NA | yes | yes | yes | NA | yes | yes |
| Korzeniewska-  Eksterowicz, 2014 | yes | yes | no | yes | NR | yes | yes | NA | yes | yes | yes | NA | yes | no |
| Kurita, 2008 | yes | no | NR | yes | NR | yes | yes | NA | yes | yes | yes | NA | yes | yes |
| Kurita, 2011 | yes | yes | yes | yes | NR | yes | yes | yes | yes | yes | yes | NA | yes | yes |
| Kurita, 2015 | yes | yes | yes | yes | NR | yes | yes | yes | yes | yes | yes | NA | yes | yes |
| Li, 2010 | yes | yes | yes | yes | NR | yes | yes | yes | yes | yes | yes | NA | yes | yes |
| Liang, 2008 | yes | no | NR | yes | NR | yes | yes | NA | yes | yes | yes | NA | yes | yes |
| Liang, 2013 | yes | no | NR | yes | NR | yes | yes | NA | yes | yes | yes | NA | yes | yes |
| Lin, 2011 | yes | yes | NR | yes | NR | yes | yes | NA | yes | yes | yes | NA | yes | yes |
| Makimura, 2011 | yes | yes | yes | yes | NR | yes | yes | yes | yes | yes | yes | NA | yes | no |
| Mercadante, 1992 | yes | no | yes | yes | NR | yes | yes | NA | yes | yes | yes | NA | yes | no |
| Mercadante, 1997 | yes | no | no | yes | NR | yes | yes | NA | yes | yes | yes | NA | yes | yes |
| Mercadante, 1998 | yes | no | NR | yes | NR | yes | yes | NA | yes | yes | yes | NA | yes | yes |
| Mercadante, 1999 | yes | no | NR | yes | NR | yes | yes | NA | yes | yes | yes | NA | yes | no |
| Mercadante, 2000a | yes | yes | NR | yes | NR | yes | yes | yes | yes | yes | yes | NA | yes | yes |
| Mercadante, 2000b | yes | no | no | yes | NR | yes | yes | NA | yes | yes | yes | NA | yes | yes |
| Mercadante, 2006 | yes | no | yes | yes | NR | yes | yes | NA | yes | yes | yes | NA | yes | no |
| Mercadante, 2009 | yes | no | NR | yes | NR | yes | yes | NA | yes | yes | yes | NA | yes | yes |
| Mercadante, 2011a | yes | no | yes | yes | NR | yes | yes | NA | yes | yes | yes | NA | yes | yes |
| Mercadante, 2011b | yes | no | yes | yes | NR | yes | yes | yes | yes | yes | yes | NA | yes | yes |
| Mercadante, 2012 | yes | yes | NR | yes | NR | yes | yes | NA | yes | yes | yes | NA | yes | no |
| Miura, 2014 | yes | yes | yes | yes | NR | yes | yes | yes | yes | yes | yes | NA | yes | yes |
| Morita, 1999 | yes | yes | NR | yes | NR | yes | yes | yes | yes | yes | yes | NA | yes | yes |
| Naito, 2012 | yes | no | NR | yes | NR | yes | yes | NA | yes | yes | yes | NA | yes | yes |
| Novy, 2012 | yes | no | yes | yes | NR | yes | yes | NA | yes | yes | yes | NA | yes | yes |
| Ozalp, 2003 | yes | no | NR | yes | NR | yes | yes | NA | yes | yes | yes | NA | yes | yes |
| Parsons, 2008 | yes | yes | yes | yes | NR | yes | yes | NA | yes | yes | yes | NA | yes | yes |
| Pina, 2015 | yes | yes | yes | yes | NR | yes | yes | NA | yes | yes | yes | NA | yes | yes |
| Radha Krishna, 2010 | yes | yes | NR | yes | NR | yes | yes | NA | yes | yes | yes | NA | yes | yes |
| Rees, 1990 | yes | yes | NR | yes | NR | yes | yes | yes | yes | yes | yes | NA | yes | yes |
| Riley, 2006 | yes | no | NR | yes | NR | yes | yes | NA | yes | yes | yes | NA | yes | yes |
| Ripamonti, 2009 | yes | yes | yes | yes | NR | yes | yes | yes | yes | yes | yes | NA | yes | yes |
| Salminen, 2013 | yes | yes | yes | yes | NR | yes | yes | NA | yes | yes | yes | NA | yes | yes |
| Stromgren, 2004 | yes | yes | yes | yes | NR | yes | yes | yes | yes | yes | yes | NA | yes | yes |
| Syrjala, 1995 | yes | no | NR | yes | NR | yes | yes | yes | yes | yes | yes | NA | yes | yes |
| Takase, 2011 | yes | yes | NR | yes | NR | yes | yes | yes | yes | yes | yes | NA | yes | yes |
| Utsumi, 2015 | yes | yes | yes | yes | NR | yes | yes | yes | yes | yes | yes | NA | yes | yes |
| Viganò, 1998 | yes | yes | yes | yes | NR | yes | yes | yes | yes | yes | yes | NA | yes | yes |
| Zyczkowska, 2013 | yes | yes | yes | yes | NR | yes | yes | yes | yes | yes | yes | NA | yes | yes |

**Reference**

- 1. Anghelescu DL, Snaman JM, Trujillo L, Sykes AD, Yuan Y, Baker JN: Patient-controlled analgesia at the end of life at a pediatric oncology institution. Pediatric blood & cancer 2015, 62(7):1237-1244.
  2. Anghelescu DL, Zhang K, Faughnan LG, Pei D: The Safety and Effectiveness of Patient-controlled Analgesia in Outpatient Children and Young Adults With Cancer: A Retrospective Study. Journal of pediatric hematology/oncology 2015, 37(5):378-382.
  3. Appelgren L, Nordborg C, Sjoberg M, Karlsson PA, Nitescu P, Curelaru I: Spinal epidural metastasis: implications for spinal analgesia to treat "refractory" cancer pain. Journal of pain and symptom management 1997, 13(1):25-42.
  4. Arthur J, Yennurajalingam S, Nguyen L, Tanco K, Chisholm G, Hui D, Bruera E: The routine use of the Edmonton Classification System for Cancer Pain in an outpatient supportive care center. Palliative & supportive care 2015, 13(5):1185-1192.
  5. Bercovitch M, Adunsky A: High dose controlled-release oxycodone in hospice care. Journal of pain & palliative care pharmacotherapy 2006, 20(4):33-39.
  6. Bercovitch M, Adunsky A: Patterns of high-dose morphine use in a home-care hospice service: should we be afraid of it? Cancer 2004, 101(6):1473-1477.
  7. Bercovitch M, Waller A, Adunsky A: High dose morphine use in the hospice setting. A database survey of patient characteristics and effect on life expectancy. Cancer 1999, 86(5):871-877.
  8. Bergman P, Sperneder S, Hoijer J, Bergqvist J, Bjorkhem-Bergman L: Low vitamin D levels are associated with higher opioid dose in palliative cancer patients--results from an observational study in Sweden. PLoS One 2015, 10(5):e0128223.
  9. Bruera E, Schoeller T, Wenk R, MacEachern T, Marcelino S, Hanson J, Suarez-Almazor M: A prospective multicenter assessment of the Edmonton staging system for cancer pain. Journal of pain and symptom management 1995, 10(5):348-355.
  10. Candrilli SD, Davis KL, Iyer S: Impact of constipation on opioid use patterns, health care resource utilization, and costs in cancer patients on opioid therapy. Journal of pain & palliative care pharmacotherapy 2009, 23(3):231-241.
  11. Cherny NI, Thaler HT, Friedlander-Klar H, Lapin J, Foley KM, Houde R, Portenoy RK: Opioid responsiveness of cancer pain syndromes caused by neuropathic or nociceptive mechanisms: a combined analysis of controlled, single-dose studies. Neurology 1994, 44(5):857-861.
  12. Chow E, Connolly R, Wong R, Franssen E, Fung KW, Harth T, Pach B, Andersson L, Schueller T, Stefaniuk K et al: Use of the CAGE questionnaire for screening problem drinking in an out-patient palliative radiotherapy clinic. Journal of pain and symptom management 2001, 21(6):491-497.
  13. Cohen MR, Pickar D, Dubois M, Bunney WE, Jr.: Stress-induced plasma beta-endorphin immunoreactivity may predict postoperative morphine usage. Psychiatry research 1982, 6(1):7-12.
  14. Collin E, Poulain P, Gauvain-Piquard A, Petit G, Pichard-Leandri E: Is disease progression the major factor in morphine 'tolerance' in cancer pain treatment? Pain 1993, 55(3):319-326.
  15. De Conno F, Groff L, Brunelli C, Zecca E, Ventafridda V, Ripamonti C: Clinical experience with oral methadone administration in the treatment of pain in 196 advanced cancer patients. Journal of clinical oncology : official journal of the American Society of Clinical Oncology 1996, 14(10):2836-2842.
  16. Dougherty M, DeBaun MR: Rapid increase of morphine and benzodiazepine usage in the last three days of life in children with cancer is related to neuropathic pain. The Journal of pediatrics 2003, 142(4):373-376.
  17. Droney J, Ross J, Gretton S, Welsh K, Sato H, Riley J: Constipation in cancer patients on morphine. Supportive care in cancer : official journal of the Multinational Association of Supportive Care in Cancer 2008, 16(5):453-459.
  18. Edrington JM, Paul S, Dodd M, West C, Facione N, Tripathy D, Koo P, Schumacher K, Miaskowski C: No evidence for sex differences in the severity and treatment of cancer pain. Journal of pain and symptom management 2004, 28(3):225-232.
  19. Fainsinger RL, Nekolaichuk C, Lawlor P, Hagen N, Bercovitch M, Fisch M, Galloway L, Kaye G, Landman W, Spruyt O et al: An international multicentre validation study of a pain classification system for cancer patients. European journal of cancer (Oxford, England : 1990) 2010, 46(16):2896-2904.
  20. Fainsinger RL, Nekolaichuk CL, Lawlor PG, Neumann CM, Hanson J, Vigano A: A multicenter study of the revised Edmonton Staging System for classifying cancer pain in advanced cancer patients. Journal of pain and symptom management 2005, 29(3):224-237.
  21. Fallon MT, Hanks GW: Morphine, constipation and performance status in advanced cancer patients. Palliative medicine 1999, 13(2):159-160.
  22. Flogegard H, Ljungman G: Characteristics and adequacy of intravenous morphine infusions in children in a paediatric oncology setting. Medical and pediatric oncology 2003, 40(4):233-238.
  23. Flynn BC, Nemergut EC: Postoperative nausea and vomiting and pain after transsphenoidal surgery: a review of 877 patients. Anesthesia and analgesia 2006, 103(1):162-167, table of contents.
  24. Gagnon P, Allard P, Masse B, DeSerres M: Delirium in terminal cancer: a prospective study using daily screening, early diagnosis, and continuous monitoring. Journal of pain and symptom management 2000, 19(6):412-426.
  25. Glare P, Walsh D, Sheehan D: The adverse effects of morphine: a prospective survey of common symptoms during repeated dosing for chronic cancer pain. The American journal of hospice & palliative care 2006, 23(3):229-235.
  26. Glare PA, Walsh TD: Dose-ranging study of oxycodone for chronic pain in advanced cancer. Journal of clinical oncology : official journal of the American Society of Clinical Oncology 1993, 11(5):973-978.
  27. Goksu SS, Bozcuk H, Uysal M, Ulukal E, Ay S, Karasu G, Soydas T, Coskun HS, Ozdogan M, Savas B: Determinants of opioid efficiency in cancer pain: a comprehensive multivariate analysis from a tertiary cancer centre. Asian Pacific journal of cancer prevention : APJCP 2014, 15(21):9301-9305.
  28. Greco MT, Corli O, Montanari M, Deandrea S, Zagonel V, Apolone G: Epidemiology and pattern of care of breakthrough cancer pain in a longitudinal sample of cancer patients: results from the Cancer Pain Outcome Research Study Group. The Clinical journal of pain 2011, 27(1):9-18.
  29. Hagen NA, Fisher K, Victorino C, Farrar JT: A titration strategy is needed to manage breakthrough cancer pain effectively: observations from data pooled from three clinical trials. Journal of palliative medicine 2007, 10(1):47-55.
  30. Hall S, Gallagher RM, Gracely E, Knowlton C, Wescules D: The terminal cancer patient: effects of age, gender, and primary tumor site on opioid dose. Pain medicine (Malden, Mass) 2003, 4(2):125-134.
  31. Hwang SS, Chang VT, Fairclough DL, Kasimis B: Development of a cancer pain prognostic scale. Journal of pain and symptom management 2002, 24(4):366-378.
  32. Kaiko RF, Wallenstein SL, Rogers AG, Houde RW: Sources of variation in analgesic responses in cancer patients with chronic pain receiving morphine. Pain 1983, 15(2):191-200.
  33. Kaiko RF: Age and morphine analgesia in cancer patients with postoperative pain. Clinical pharmacology and therapeutics 1980, 28(6):823-826.
  34. Kanbayashi Y, Hosokawa T, Okamoto K, Fujimoto S, Konishi H, Otsuji E, Yoshikawa T, Takagi T, Miki T, Taniwaki M: Factors predicting requirement of high-dose transdermal fentanyl in opioid switching from oral morphine or oxycodone in patients with cancer pain. The Clinical journal of pain 2011, 27(8):664-667.
  35. Karavelis A, Foroglou G, Selviaridis P, Fountzilas G: Intraventricular administration of morphine for control of intractable cancer pain in 90 patients. Neurosurgery 1996, 39(1):57-61; discussion 61-52.
  36. Knudsen AK, Brunelli C, Kaasa S, Apolone G, Corli O, Montanari M, Fainsinger R, Aass N, Fayers P, Caraceni A et al: Which variables are associated with pain intensity and treatment response in advanced cancer patients?--Implications for a future classification system for cancer pain. European journal of pain (London, England) 2011, 15(3):320-327.
  37. Knudsen AK, Brunelli C, Klepstad P, Aass N, Apolone G, Corli O, Montanari M, Caraceni A, Kaasa S: Which domains should be included in a cancer pain classification system? Analyses of longitudinal data. Pain 2012, 153(3):696-703.
  38. Kurita GP, de Mattos Pimenta CA: Cognitive impairment in cancer pain patients receiving opioids: a pilot study. Cancer nursing 2008, 31(1):49-57.
  39. Kurita GP, Lundstrom S, Sjogren P, Ekholm O, Christrup L, Davies A, Kaasa S, Klepstad P, Dale O: Renal function and symptoms/adverse effects in opioid-treated patients with cancer. Acta anaesthesiologica Scandinavica 2015, 59(8):1049-1059.
  40. Kurita GP, Sjogren P, Ekholm O, Kaasa S, Loge JH, Poviloniene I, Klepstad P: Prevalence and predictors of cognitive dysfunction in opioid-treated patients with cancer: a multinational study. Journal of clinical oncology : official journal of the American Society of Clinical Oncology 2011, 29(10):1297-1303.
  41. Li M-H YE-T, Huang S-C, Wang H-M, Su W-R, Lai Y-L: Clinical Experience With Strong Opioids in Pain Control of Terminally ill Cancer Patients in Palliative Care Settings in Taiwan. Journal of Experimental and Clinical Medicine 2010, 2(6):292-296.
  42. Liang SY, Yates P, Edwards H, Tsay SL: Factors influencing opioid-taking self-efficacy and analgesic adherence in Taiwanese outpatients with cancer. Psycho-oncology 2008, 17(11):1100-1107.
  43. Lin YL, Lin IC, Liou JC: Symptom patterns of patients with head and neck cancer in a palliative care unit. Journal of palliative medicine 2011, 14(5):556-559.
  44. Makimura C, Arao T, Matsuoka H, Takeda M, Kiyota H, Tsurutani J, Fujita Y, Matsumoto K, Kimura H, Otsuka M et al: Prospective study evaluating the plasma concentrations of twenty-six cytokines and response to morphine treatment in cancer patients. Anticancer research 2011, 31(12):4561-4568.
  45. Mercadante S, Casuccio A, Agnello A, Barresi L: Methadone response in advanced cancer patients with pain followed at home. Journal of pain and symptom management 1999, 18(3):188-192.
  46. Mercadante S, Casuccio A, Pumo S, Fulfaro F: Factors influencing the opioid response in advanced cancer patients with pain followed at home: the effects of age and gender. Supportive care in cancer : official journal of the Multinational Association of Supportive Care in Cancer 2000, 8(2):123-130.
  47. Mercadante S, Casuccio A, Pumo S, Fulfaro F: Opioid responsiveness-primary diagnosis relationship in advanced cancer patients followed at home. Journal of pain and symptom management 2000, 20(1):27-34.
  48. Mercadante S, Dardanoni G, Salvaggio L, Armata MG, Agnello A: Monitoring of opioid therapy in advanced cancer pain patients. Journal of pain and symptom management 1997, 13(4):204-212.
  49. Mercadante S, Ferrera P, Casuccio A: Prevalence of opioid-related dysuria in patients with advanced cancer having pain. The American journal of hospice & palliative care 2011, 28(1):27-30.
  50. Mercadante S, Ferrera P, David F, Casuccio A: The use of high doses of oxycodone in an acute palliative care unit. The American journal of hospice & palliative care 2011, 28(4):242-244.
  51. Mercadante S, Ferrera P, Villari P, Casuccio A, Intravaia G, Mangione S: Frequency, indications, outcomes, and predictive factors of opioid switching in an acute palliative care unit. Journal of pain and symptom management 2009, 37(4):632-641.
  52. Mercadante S, Ferrera P, Villari P, Casuccio A: Opioid escalation in patients with cancer pain: the effect of age. Journal of pain and symptom management 2006, 32(5):413-419.
  53. Mercadante S, Maddaloni S, Roccella S, Salvaggio L: Predictive factors in advanced cancer pain treated only by analgesics. Pain 1992, 50(2):151-155.
  54. Mercadante S: Opioid responsiveness in patients with advanced head and neck cancer. Supportive care in cancer : official journal of the Multinational Association of Supportive Care in Cancer 1998, 6(5):482-485.
  55. Mercadante S: Switching methadone: a 10-year experience of 345 patients in an acute palliative care unit. Pain medicine (Malden, Mass) 2012, 13(3):399-404.
  56. Morita T, Tsunoda J, Inoue S, Chihara S: Contributing factors to physical symptoms in terminally-ill cancer patients. Journal of pain and symptom management 1999, 18(5):338-346.
  57. Naito T, Tashiro M, Yamamoto K, Ohnishi K, Kagawa Y, Kawakami J: Impact of cachexia on pharmacokinetic disposition of and clinical responses to oxycodone in cancer patients. European journal of clinical pharmacology 2012, 68(10):1411-1418.
  58. Novy DM, Lam C, Gritz ER, Hernandez M, Driver LC, Koyyalagunta D: Distinguishing features of cancer patients who smoke: pain, symptom burden, and risk for opioid misuse. The journal of pain : official journal of the American Pain Society 2012, 13(11):1058-1067.
  59. Ozalp G, Sarioglu R, Tuncel G, Aslan K, Kadiogullari N: Preoperative emotional states in patients with breast cancer and postoperative pain. Acta anaesthesiologica Scandinavica 2003, 47(1):26-29.
  60. Park JE, Kim KI, Yoon SS, Hahm BJ, Lee SM, Yoon JH, Shin WG, Lee HS, Oh JM: Psychological distress as a negative survival factor for patients with hematologic malignancies who underwent allogeneic hematopoietic stem cell transplantation. Pharmacotherapy 2010, 30(12):1239-1246.
  61. Parsons HA, Delgado-Guay MO, El Osta B, Chacko R, Poulter V, Palmer JL, Bruera E: Alcoholism screening in patients with advanced cancer: impact on symptom burden and opioid use. Journal of palliative medicine 2008, 11(7):964-968.
  62. Pickar D, Cohen MR, Dubois M: The relationship of plasma cortisol and beta-endorphin immunoreactivity to surgical stress and postoperative analgesic requirement. General hospital psychiatry 1983, 5(2):93-98.
  63. Pina P, Sabri E, Lawlor PG: Characteristics and associations of pain intensity in patients referred to a specialist cancer pain clinic. Pain research & management : the journal of the Canadian Pain Society = journal de la societe canadienne pour le traitement de la douleur 2015, 20(5):249-254.
  64. Radha Krishna LK, Poulose JV, Tan BS, Goh C: Opioid use amongst cancer patients at the end of life. Annals of the Academy of Medicine, Singapore 2010, 39(10):790-797.
  65. Rees WD: Opioid needs of terminal care patients: variations with age and primary site. Clinical oncology (Royal College of Radiologists (Great Britain)) 1990, 2(2):79-83.
  66. Riley J, Ross JR, Rutter D, Wells AU, Goller K, du Bois R, Welsh K: No pain relief from morphine? Individual variation in sensitivity to morphine and the need to switch to an alternative opioid in cancer patients. Supportive care in cancer : official journal of the Multinational Association of Supportive Care in Cancer 2006, 14(1):56-64.
  67. Ripamonti CI, Campa T, Fagnoni E, Brunelli C, Luzzani M, Maltoni M, De Conno F: Normal-release oral morphine starting dose in cancer patients with pain. The Clinical journal of pain 2009, 25(5):386-390.
  68. Salminen EK, Silvoniemi M, Syrjanen K, Kaasa S, Kloke M, Klepstad P: Opioids in pain management of mesothelioma and lung cancer patients. Acta oncologica (Stockholm, Sweden) 2013, 52(1):30-37.
  69. Stromgren AS, Groenvold M, Petersen MA, Goldschmidt D, Pedersen L, Spile M, Irming-Pedersen G, Sjogren P: Pain characteristics and treatment outcome for advanced cancer patients during the first week of specialized palliative care. Journal of pain and symptom management 2004, 27(2):104-113.
  70. Syrjala KL, Chapko ME: Evidence for a biopsychosocial model of cancer treatment-related pain. Pain 1995, 61(1):69-79.
  71. Takase H, Sakata T, Yamano T, Sueta T, Nomoto S, Nakagawa T: Advantage of early induction of opioid to control pain induced by irradiation in head and neck cancer patients. Auris, nasus, larynx 2011, 38(4):495-500.
  72. Vigano A, Bruera E, Suarez-Almazor ME: Age, pain intensity, and opioid dose in patients with advanced cancer. Cancer 1998, 83(6):1244-1250.
  73. Yang JC, Clark WC, Tsui SL, Ng KF, Clark SB: Preoperative Multidimensional Affect and Pain Survey (MAPS) scores predict postcolectomy analgesia requirement. The Clinical journal of pain 2000, 16(4):314-320.
  74. Życzkowska J GT, Kleja J, Filipczak-Bryniarska I, Wrzosek A, Wordliczek J: Age influence on opioid consumption in terminally ill digestive cancer patients. Medycyna Paliatywna w Praktyce 2013, 7(2):50-54.
